# Sustainable synthesis of microwave-assisted IONPs using *Spinacia oleracea* L. for control of fungal wilt by modulating the defense system in tomato plants

**DOI:** 10.1186/s12951-021-01204-9

**Published:** 2022-01-04

**Authors:** Hina Ashraf, Tehmina Anjum, Saira Riaz, Tanzeela Batool, Shahzad Naseem, Guihua Li

**Affiliations:** 1 Guangdong Key Laboratory for New Technology Research of Vegetables/Vegetable Research Institute, Guangdong Academy of Agricultural Sciences, Guangzhou, People’s Republic of China; 2 Department of Plant Pathology, Faculty of Agricultural Sciences, University of the Punjab, Lahore, Pakistan; 3 Centre of Excellence in Solid-State-Physics, University of the Punjab, Lahore, Pakistan

**Keywords:** Tomato, Fusarium wilt, IONPs, Microwave, Green synthesis, Anti-oxidant enzymes, ROS, Greenhouse

## Abstract

**Background:**

Changing climate enhances the survival of pests and pathogens, which eventually affects crop yield and reduces its economic value. Novel approaches should be employed to ensure sustainable food security. Nano-based agri-chemicals provide a distinctive mechanism to increase productivity and manage phytopathogens, with minimal environmental distress. In vitro and in greenhouse studies were conducted to evaluate the potential of green-synthesized iron-oxide nanoparticles (IONPs) in suppressing wilt infection caused by *Fusarium oxysporum* f. sp. *lycospersici*, and improving tomato growth (*Solanum lycopersicum*) and fruit quality.

**Results:**

Various microwave powers (100–1000 W) were used to modulate the properties of the green-synthesized IONPs, using spinach as a starting material. The IONPs stabilized with black coffee extract were substantively characterized using X-ray diffraction analysis (XRD), Fourier-transform infrared spectroscopy, dielectric and impedance spectroscopy, X-ray photoelectron spectroscopy (XPS), scanning and transmission electron microscopy (SEM and TEM, respectively), and magnetization analysis. XRD revealed a cubic magnetite (Fe_3_O_4_) phase with super-paramagnetic nature, detected at all microwave powers. The binding energies of Fe 2p_3/2_ (710.9 eV) and Fe 2p_1/2_ (724.5 eV) of Fe_3_O_4_ NPs were confirmed using XPS analysis at a microwave power of 1000 W. Uniform, spherical/cubical-shaped particles with an average diameter of 4 nm were confirmed using SEM and TEM analysis. A significant reduction in mycelial growth and spore germination was observed upon exposure to different IONP treatments. Malformed mycelium, DNA fragmentation, alternation in the cell membrane, and ROS production in *F. oxysporum* indicated the anti-microbial potential of the IONPs. The particles were applied both through the root (before transplantation) and by means of foliar application (after two weeks) to the infected seedlings. IONPs significantly reduced disease severity by an average of 47.8%, resulting in increased plant growth variables after exposure to 12.5 µg/mL of IONPs. Analysis of photosynthetic pigments, phenolic compounds, and anti-oxidant enzymes in the roots and shoots showed an increasing trend after exposure to various concentrations of IONPs. Correspondingly, lycopene, vitamin C, total flavonoids, and protein content were substantially improved in tomato fruits after treatment with IONPs.

**Conclusion:**

The findings of the current investigation suggested that the synthesized IONPs display anti-fungal and nutritional properties that can help to manage Fusarium wilt disease, resulting in enhanced plant growth and fruit quality.

**Graphical Abstract:**

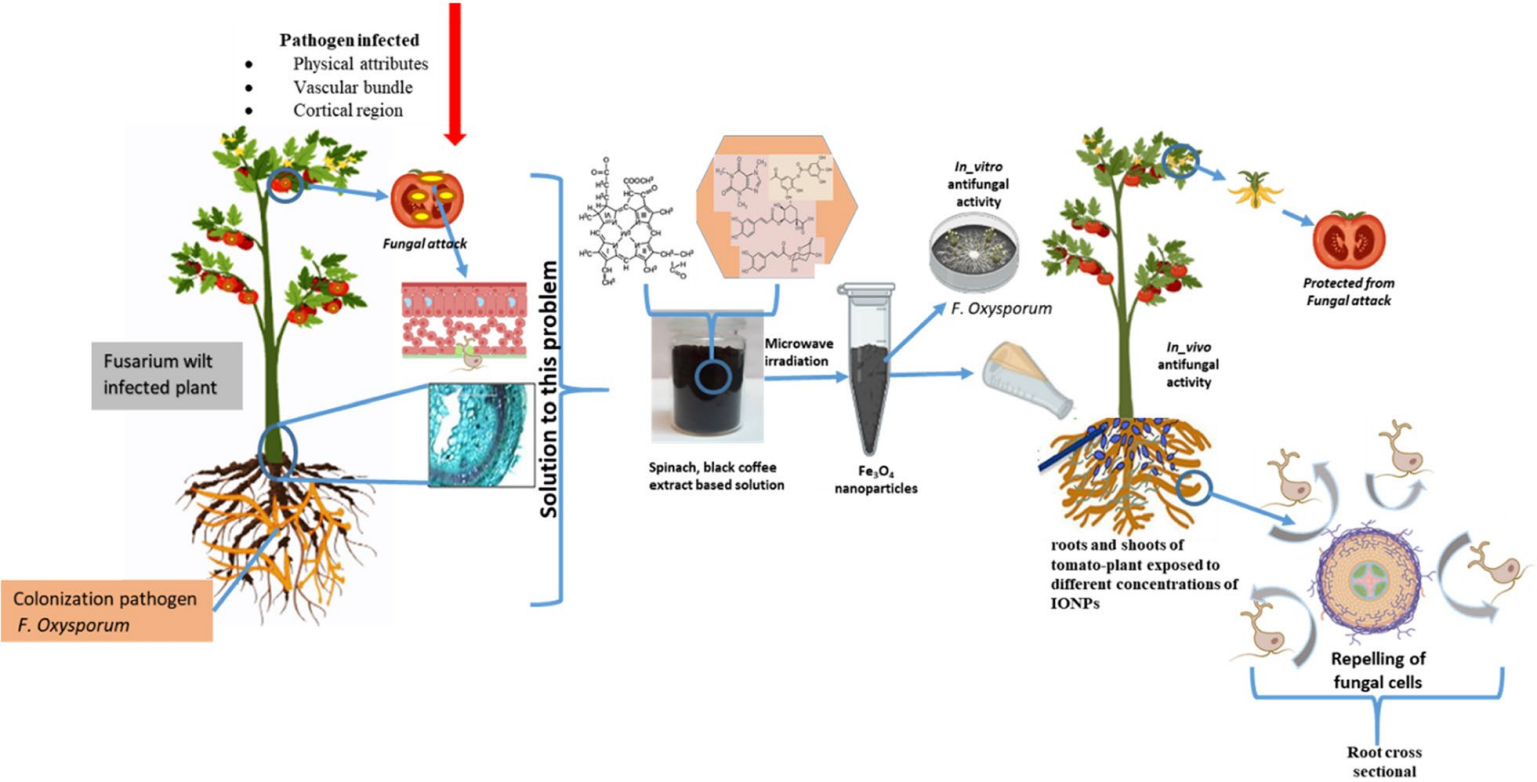

**Supplementary Information:**

The online version contains supplementary material available at 10.1186/s12951-021-01204-9.

## Background

Innovative and advanced technological approaches have recently been employed in the agricultural sector, to encounter the surging challenges of food security and sustainable production [1]. Stratagems based on “nanoparticle-technology” have resulted in fascinating outputs owing to their innate characteristics [2]. Novel practices in agriculture are a dynamic factor in research, to meet the needs for consistent food supply to the global population [3]. Nanotechnology, an emerging interdisciplinary field of the modern era, has an important impact on people’s lives, as it has the potential to resolve most of the scientific problems, and thus, has proven its essentiality in most fields, including agriculture and allied industries [4]. Nanoscale materials, owing to their distinctive properties, such as improved efficacy, lower eco-toxicity, and reduced inputs, provide an auspicious alternative for crop protection that has numerous advantages over traditional approaches and products [5]. Among the various classes of nanoparticles (NPs), metal-oxide nanomaterials are considered innocuous for both the environment and humans. Earlier studies have indicated the biocompatibility of iron-oxide nanoparticles (IONPs), which possess anti-microbial properties against various pathogenic fungi and bacteria [6]. Nowadays, nanotechnology has a new function in agriculture, in bringing about the suppression of pathogen infection (bacterial, viral, and fungal), by improving plant nutrition, and thus, directly enhancing nutritional value and crop yield [7]. Additionally, nanomaterials may affect plant cells and their developmental stages, including seed germination, root induction, cell metabolism, their growth index and biomass, as well as, alter their redox levels [8]. Iron is a pivotal micronutrient cognate with various physiological functions in plants, but it is generally found in an insoluble Fe^+3^ form. Owing to the low solubility of minerals containing iron, one method to overcome iron deficiency is the use of NPs either as iron or as IONPs [9]. IONPs are reduced in size and have higher solubility than other complex molecules, which facilitates plants with a greater level of iron [10]. Therefore, these compounds provide an optimum environment for plant enhancement and aversion of stress conditions, by generating secondary metabolites [11]. Iron is a key constituent of cell redox reactions that serves as a co-factor for various anti-oxidant enzymes, such as catalase (CAT), superoxide dismutase (SOD), and peroxidase (POD), and as a scavenger of reactive oxygen species (ROS) [12]. Although oxidative stress is induced in response to NPs, these trigger the defense mechanism in plants, such as higher activities of anti-oxidant enzymes, which eventually scavenge the ROS [13]. Earlier reports have indicated that under stress conditions, IONPs elevate the anti-oxidant activity in various plants such as *Mentha piperita* [9], *Triticum aestivum* [14, 15], *Solanum lycopersicum* [10], and *Dracocephalum moldavica* [4].

Tomato (*Solanum lycopersicum* L.) is the most notable crop after potato, from a commercial and economic perspective, and is ranked sixth in the world by the Food and Agriculture Organization, in terms of total annual production [16]. However, its crop yield is potentially reduced to 20% due to soil-borne diseases that are difficult to cope with. Alone, fungal pathogens are responsible for the loss of millions per year, by reducing the economic return [17]. Fusarium wilt, one of the most devastating fungal diseases that diminishes the yield index and nutritive value of various crops, particularly tomatoes, is initiated by *Fusarium oxysporum* f. sp. *lycopersici*, which affects tomato growth equally under greenhouse and field trials [18]. Although there are different traditional ways to overcome this disease, such as the use of resistant varieties and application of fungicides, these approaches are environmentally unsustainable and cost ineffectual [17]. Thus, there is a demand for more innovative and effective ways to cope with fungal pathogens. A limited number of reports are available on the role of IONPs in disease management. Alam et al*.* reported the anti-fungal efficacy of green-synthesized IONPs against the polyphagous pathogen *Verticillium dahlia*, by preventing the proliferation of mycelium [19]. Fe_3_O_4_ NPs induced the expression of miR159c in yellow medicinal plants against powdery mildew [20]. In vitro application of IONPs showed significant anti-fungal properties, inhibiting spore germination in various phytopathogens, such as *Aspergillus niger* and *Fusarium solani* [21], *Rhizopus oryzae* [22], *Trichothecium roseum*, *Cladosporium herbarum*, *Penicillium chrysogenum*, *Alternaria alternata*, and *A. niger* [23], *P. expansum, A. niger*, *A. alternata*, *P. chrysogenum*, *Mucor plumbeus*, *T. roseum*, and *Rhizoctonia solani* [24]. Earlier studies indicated that the formation and accumulation of ROS in microbial cells impedes the multiplication ability of the pathogens, thus depicting the underlying mechanism of metal NPs. However, it is commonly anticipated that the anti-microbial activity relies on direct interactions between the NPs and living cells [25]. Magnesium-oxide NPs have revealed antagonistic activity against fungal species by acting directly on fungal cells [26]. Chitosan-coated IONPs showed significant anti-microbial activity against *Escherichia coli* and *Bacillus subtilis*, by upregulating the ROS levels [27].

Tomatoes are an important part of the human diet as they contain various bioactive compounds such as total phenols, lycopene, vitamin C, carotenoids, and total flavonoids, which act as anti-oxidant compounds with specific physicochemical and biological properties and play a beneficial role in human health [28]. As IONPs are considered safe for use in medical and food applications and are permitted by the Food and Drug Administration (FDA) [29], they can also be explored as an environmentally safe alternative to synthetic fungicides in the agriculture sector.

In the current investigation, it was hypothesized that green-synthesized IONPs would be more efficient in suppressing Fusarium wilt disease in infected tomato plants. We demonstrated the synthesis of IONPs by utilizing spinach powder and black coffee (BC) extract at various microwave powers from 100 to 1000 W. The findings of this study indicated the improved efficacy of microwave-assisted IONPs in subduing fungal growth and enhancing plant growth, under both in vitro and in vivo conditions. In addition, alterations in mycelia, ROS production, membrane integrity, and DNA fragmentation were evaluated in a fungal pathogen. Importantly, the effects of IONPs on plant germination, disease index, and enzymatic and non-enzymatic anti-oxidant enzymes were also measured. These findings also provided significant information on the activation of defensive pathways in tomato plants and their potential to replace traditional fertilizers or fungicides. Furthermore, these outcomes can serve as a basis for the design of nano-based tools for sustainable agriculture.

## Materials and methods

### Materials, reagents, and fungal culture

All reagents and chemicals were of analytical grade, obtained from Sigma-Aldrich (St Louis, MO, USA), and consumed without any further purification, unless specified otherwise. Fresh spinach leaves (*Spinacia oleracea*), purchased from the local vegetable market of Lahore, Pakistan, were used as the starting material. BC extract was prepared by dissolving 20 g of coffee powder in 100 mL of deionized water (DI water) and then adjusting the pH to 5. Tomato seeds were obtained from the Ayub Agriculture Research Institute (AARI), Faisalabad, Pakistan. *“Fusarium oxysporum* f. sp. *lycopersici* (IAGS-1322)” used in this investigation as a challenging tomato pathogen was issued by Fungal-Biotechnology-Lab, Department of Plant Pathology, Faculty of Agricultural Sciences, University of the Punjab, Lahore; it was previously isolated from infected tomato fields and was preferred in this study, based on its virulence. The stock culture was grown and maintained on potato dextrose agar (PDA) slants at 4 °C for long-term use. For solid cultures of *F. oxysporum*, stock cultures were sub-cultured onto petri-plates containing PDA and incubated for 7 d in the dark at 28 °C. DI water was used as the solvent.

### Green synthesis of IONPs

**Fig.1:**
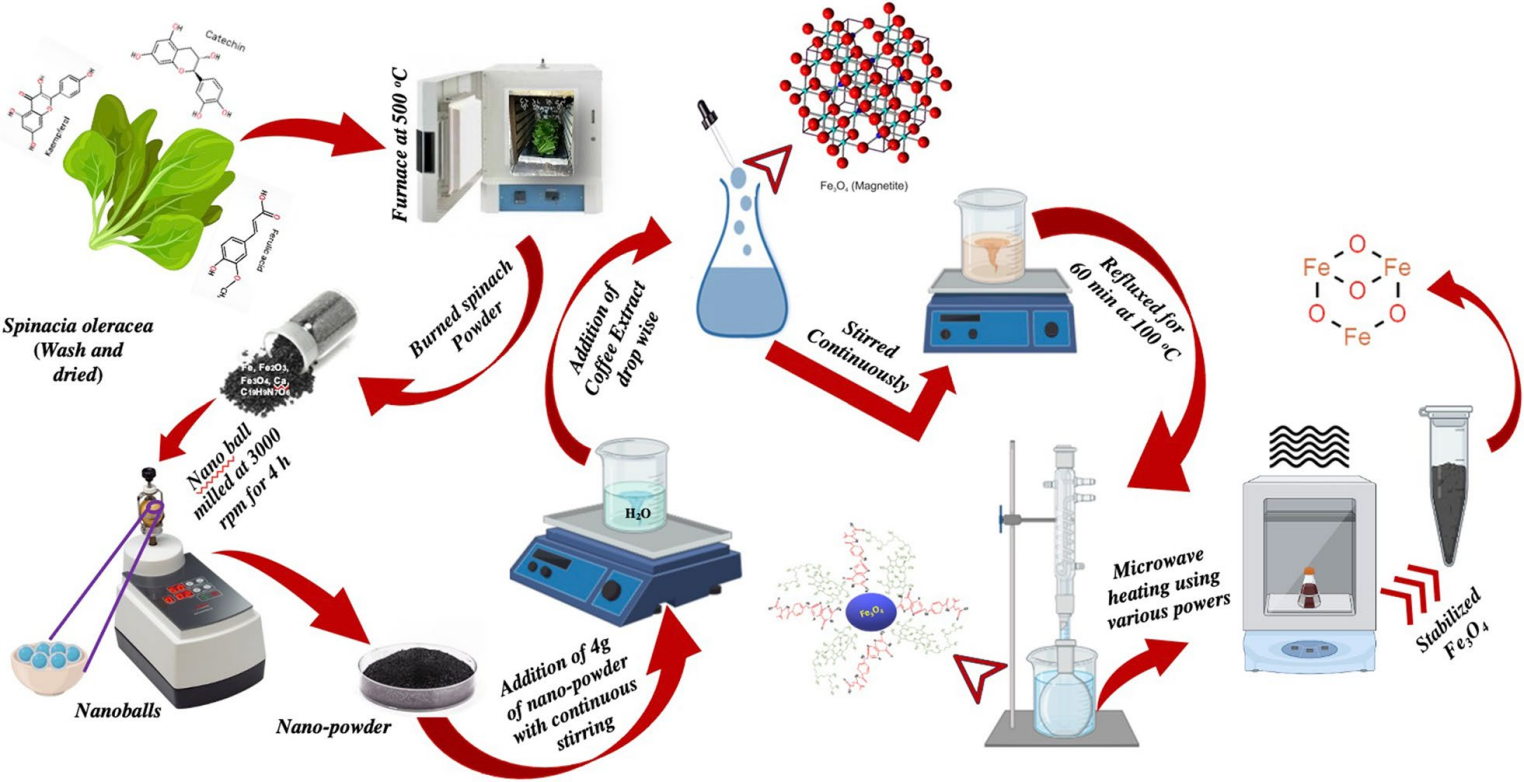
Schematic showing green synthesis of IONPs using spinach and black coffee extract by varying microwave powers (100–1000 W)

For the synthesis of IONPs, *Spinacia oleracea* was used as the starting material. Initially, spinach leaves were washed with DI water and further dried at room temperature (25 °C) for 2 h. Dried leaves were kept in a muffle furnace at 500 °C for 2 h, to obtain powdered leaves. Nano ball-milling was performed for 4 h at 3000 rpm in a pulverisette (23 mini mill, Fritsch), to obtain a fine nanopowder. Four grams of the nano-milled powder was dissolved in 400 mL of DI water under vigorous stirring (solution A). BC extract was added drop-wise to solution ‘A’, to prepare 0.01 M solution ‘B’ under continuous stirring. Solution ‘B’ was further subjected to various microwave powers, i.e., 100–1000 W, for the formation of stabilized iron-oxide powder. The on and off times of the microwave were precisely controlled to overcome the spitting process due to the localized heat generated in the microwave oven. The resultant product was separated by means of centrifugation and dried in a vacuum oven at 80 °C for further characterization. Details regarding the characterization instruments are discussed in Additional file 1: Experiment S1. A schematic of this process is shown in Fig. 1.

### Inhibitory effect of IONPs on fungal mycelial growth

The in vitro anti-fungal activity of IONPs on the mycelial growth of *F. oxysporum* was evaluated using the agar dilution protocol. Twenty milliliters of PDA was poured into sterilized Petri dishes, and a measured amount of IONPs from the stock solution was added to obtain the required concentrations. The final concentrations in the growth media were 0.01, 0.5, 1.5, 2.5, 5, 7.5, 10, 12.5, and 15 µg of IONPs per mL. PDA plates without IONPs were used as negative controls, while plates treated with fungicide served as the positive control. A fungal disc (4 mm in diameter) was incised from the fringe of a seven-day-old culture of *F. oxysporum* and inoculated aseptically at the center of each PDA solid media plate. The plates were then sealed and incubated at 28 °C until growth in the control plates extended to the periphery. Each treatment was performed in triplicates. Photographs were captured, and mycelial radial growth was estimated after seven days. The percentage of inhibition of radial growth induced by different concentrations of IONPs, in comparison to the control, was calculated using Eq. (1):

1 $$\mathrm{Percent\,Inhibition\,Radial\,Growth }\left({\%}\right)=\left[{\mathrm{R}}_{1}- {\mathrm{R}}_{2}\right] /{R}_{1}\times 100$$

where, R_**1**_: mean radial growth in the control group; R_2_: mean radial growth in the treatment group [30]. The protocol for determining the suppressive activity of IONPs on fungal spore germination is discussed in Additional file 1: Experiment S2.

### Evaluating morphological alterations in fungal mycelia using scanning electron microscopy (SEM)

The morphological and ultrastructural changes in fungal hyphae after treatment with IONPs were examined using field-emission scanning electron microscopy (FE-SEM). In brief, control and NP-treated *F. oxysporum* (10 and 15 µg/mL) mycelium were scratched out and fixed for 4 h with glutaraldehyde (2.5%) at 4 °C, post-fixed with aqueous-osmium-tetroxide (1%), following which the fixed sample was washed several times with phosphate-buffered saline (PBS pH 7, 0.5 M). The hyphal samples were then exsiccated with a gradient series of ethanol (30: 50: 70%: 80: 90: and 100%) for 20 min per series. Subsequently, the hyphal sections were left overnight in isoamyl acetate. Finally, the sections were exposed to an analytical level of dry CO_2_, conductively coated with gold sputter, and observed using FE-SEM (S-4800, Hitachi, Japan).

### Evaluating ROS production

Oxidative stress in fungal hyphae induced by IONPs was detected using a redox-sensitive fluorescent probe, 2',7'-dichloro-dihydro-fluorescein-diacetate (H_2_DCFDA), following the protocol of Chen et al*.* [26], with some modifications. Briefly, *F. oxysporum* mycelium was cultured in potato dextrose broth (PDB) supplemented with three different concentrations of IONPs (5, 10, and 15 µg/mL) for 24 h at 28 °C. Later, the hyphae were amassed by means of centrifugation for 10 min at 3500 rpm, followed by discarding of media. Subsequently, the mycelia were washed and re-suspended in PBS. The samples were then stained for 20 min with 10 µM H_2_DCFDA under dark conditions at room temperature. The percentage of intracellular ROS in the IONP-treated mycelial samples was calculated with respect to the untreated control (100%). The visual detection of ROS production in fungal mycelia was carried out using confocal laser scanning fluorescence microscopy (CLSM, LSM 7, Zeiss), with emission at 525 nm and excitation at 488 nm. Quantification of fluorescence intensity was performed using ImageJ software [31].

### Evaluation of plasma membrane integrity

The integrity of the plasma membrane of *F. oxysporum* after treatment with IONPs was explored using propidium iodide (PI: a membrane-impermeable dye), according to the protocol described by Wei et al*.* [32], with minor modifications. Fungal mycelia were collected following the protocol described above. A suspension not treated with NPs was included as a control. Subsequently, the collected mycelia were stained with 10 µg/mL PI for 15 min at 30 °C, under dark conditions. After centrifugation at 5000 rpm for 5 min at 4 °C, the mycelia were rinsed twice with 0.5 M PBS (pH 7), to eradicate residual dyes and re-suspended in the same buffer. Samples were observed using CLSM, with emission at 617 nm and excitation at 536 nm, and the results were analyzed as a percentage of the control. Quantification of fluorescence intensity was carried out using ImageJ software.

### Assessment of genomic DNA fragmentation

Fresh fungal cultures were treated with different concentrations (0.1–15 µg/mL) of IONPs, centrifuged (10 min; 12,000 rpm; 4 °C) to obtain pellets, and lysed with lysis buffer (10 mM EDTA, 0.5% Triton™ X-100, 0.1 M NaCl, 10 mM Tris–HCl, 2% SDS, pH 7.5). The mixture was then incubated for 30 min at 37 °C. Next, the lysates were incubated with RNase (0.03 mg/L) and proteinase K (0.2 mg/mL) at 55 °C for 1 h. After incubation, the mixture was centrifuged for 10 min at 10,000 rpm. The nuclear-DNA of *F. oxysporum* was isolated using a phenol–chloroform mixture. The aqueous phase was then transferred to a new tube, purified with isopropanol, and stored at − 20 °C for 20 min. The DNA samples were rinsed three times with 70% ethanol and re-suspended in TE buffer. DNA samples were run on a 1% agarose gel in 1 × TAE buffer at 100 V for 60 min and stained with ethidium bromide (1 mg/mL). The gel photographs were captured using a UV-transilluminator (Witeg, Germany).

### Evaluating the impact of IONPs using a green-house assay

A tomato variety (Rio-Grande) susceptible to wilt disease caused by *F. oxysporum* was grown in small plastic pots under controlled conditions (temperature = 28–30 ± 1 °C; humidity = 85–90%) for 25–30 d, until 3–5 leaves developed. The fungal pathogen *Fusarium oxysporum* was grown on PDB to develop a conidial suspension, while the inoculated concentration was adjusted to 1 × 10^6^ spore/mL. Specifically, one week before seedling transplantation, each plastic pot filled with sterilized soil received 30 mL of spore suspension in a separate trial. NP treatment was performed using the root-dipping method [33], by keeping tomato plant roots in various concentrations of IONPs (0.1–15 µg/mL) at room temperature. Uniform tomato seedlings were then transferred to the infected soil, with three plants per pot. Positive and negative controls were treated with fungicide (Nativo) and sterilized water. Foliar applications of NP sprays with an interim of five days were performed after two weeks of transplanting. Each treatment was adjusted to five replicates. The Fusarium wilt disease incidence and percent disease severity (PDS) were evaluated at 25 d post-inoculation. Disease severity for Fusarium wilt on tomato plants was determined using the following 0–6 indexing scale, with minor modifications [34]. Disease severity scale: (1 = immune: symptomless; 2 = resistant: initiation of wilting symptoms, 5% leaves showing yellowing and wilting; 3 = moderately resistant: 6–10% wilting and yellowing of leaves; 4 = moderately susceptible: 11–12% leaves showing symptoms; 5 = susceptible: 21–50% leaves showing wilting with yellow–brown bi-coloration; and 6 = highly susceptible: > 50% leaves showing infection, dying, and drying of plant. PDS was calculated using Eq. (2):

2 $$Disease\,Severity \left(\%\right)=\sum \left\{\left(\eta \times \mathcal{V}\right)/\left(6\times \mathrm{\rm N}\right)\right\}\times 100$$

where n is the number of diseased plants, V is the numerical grade of diseased plants, N is the total number of plants examined in each treatment, and 6 is the highest grade of infection category.

While disease incidence was calculated using Eq. (3):

3 $$Disease\,Incidence\,(\% ) = No.\,of\,infected\,plants/Total\,no.\,of\,plants \times 100$$

Methods to evaluate physiological and biochemical variations in tomato roots and shoots after treatment with different concentrations of IONPs are discussed in Additional file 1: Experiment S3. After 60 d of transplantation, tomato plants were carefully uprooted to calculate growth variables such as plant height, length (root and shoot), and plant biomass (fresh and dry weight).

### Statistical analysis

Statistical analysis was performed using Prism (8.4.3) software (GraphPad, San Diego, CA, USA). One-way analysis of variance or Student’s *t*-test was employed to compare group means, using Tukey’s multiple comparison *post-hoc* test. The outcomes have been presented as mean ± standard deviation for three or more replicates. Asterisks (*p < 0.05, **p < 0.01, and ***p < 0.001) represent significant differences.

## Results and discussion

### Structural analysis of green-synthesized IONPs

**Fig. 2:**
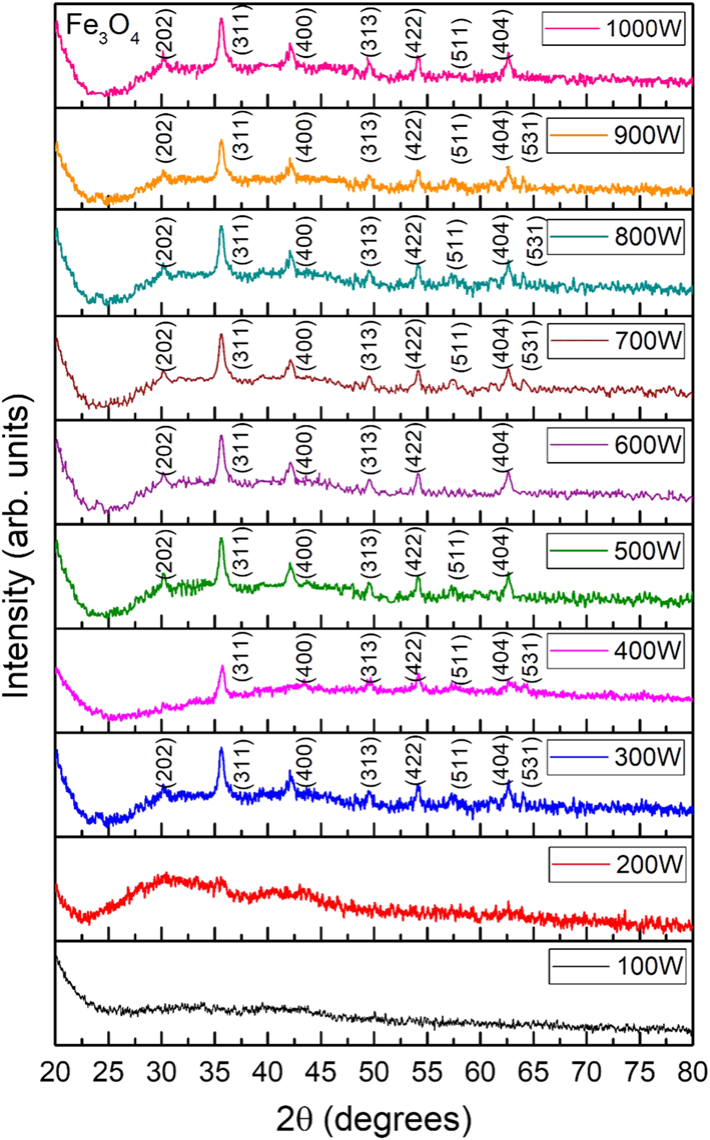
Structural analysis by X-ray diffraction: XRD spectra of Fe_3_O_4_ NPs synthesized by using spinach leaves and Black coffee (BC) extract, subjected to various microwave powers (100–1000 W) with CuKα (1.5406 Å) radiations in the range of 2θ = 20–80º and an operating voltage of 40 kV at 15 mA

Figure 2 shows the X-ray diffraction (XRD) pattern of IONPs synthesized using spinach leaves and BC extract, at various microwave powers (100–1000 W). XRD results demonstrated the presence of phase-pure spinal-structured magnetite NPs at all the microwave powers. The peaks at 2θ angles of 30.02°, 35.64°, 43.44°, 49.79°, 54.1°, 57.35°, 62.72° and 64.18° corresponded to the diffraction planes of (202), (311), (400), (313), (422), (511), (404), and (531), respectively. The peaks of the Fe_3_O_4_ NPs were indexed with JCPDS card no. 96-900-2317. In the present study, BC was used as the reducing agent. The major constituents of BC are caffeine and tannins. Tannins are composed of polyphenolic compounds (non-toxic), which are reducing and stabilizing agents for the synthesis of IONPs. Phenolic-OH and ortho-dihydroxy phenyl groups present in the chemical structure of tannins are responsible for the complex formation with iron. These groups also participate in redox reactions [35]. Therefore, the production of Fe_3_O_4_ NPs is mainly governed by the tannins present in the BC extract. In addition, the use of microwave radiation makes it possible to synthesize pure-phase Fe_3_O_4_ NPs, without any further heat treatment. Microwave energy is converted into heat energy, which highly depends on the nature of solvent utilization. The parameter that determines the capability of the material for this conversion is termed as “tangent loss” (tan δ). For microwave radiation with a typical frequency of 2.45 GHz, solvents with a higher value of tan δ are the preferred choice, as they can absorb an excellent amount of radiation and convert it into heat energy [36]. Owing to the higher tangent loss of polyphenols (i.e., constituents of BC, tannin), high heat will be produced in the microwave oven, thus leading to phase-pure Fe_3_O_4_ NPs even without further heat treatment.

The crystallite size (t) and dislocation density (δ) were plotted (Additional file 1: Fig. S1) and calculated using Eqs. (4 and 5), respectively [37].

4 $$\mathrm{t}=\frac{k\lambda }{BCos\theta }$$

5 $$\delta = {\raise0.7ex\hbox{$1$} \!\mathord{\left/ {\vphantom {1 {t^{2} }}}\right.\kern-\nulldelimiterspace} \!\lower0.7ex\hbox{${t^{2} }$}}$$

where k is the shape factor, considered as 0.9 in this case, λ is the wavelength of the Cu Kα source, β is the full width at half maximum (FWHM), and θ is the diffraction angle of the highest intensity peak. Variation in crystallite size was observed with variation in microwave power. BC, along with microwave heating, played a critical role not only in achieving the Fe_3_O_4_ phase of iron-oxide (Fig. 2), but also in achieving a small crystallite size (Additional file 1: Fig. S1). XRD analysis also revealed that the NPs synthesized using 100–200 W power possessed amorphous behavior, while at the higher microwave powers, a crystalline nature of IONPs was observed, along with phase stability (Fig. 2). The major factors influencing the crystallite size are the lowest surface energy, grain boundary energy, and diffusion of surface atoms. Microwave energy was observed to be effective in tuning these parameters, thus leading to the formation of stabilized and pure-phased Fe_3_O_4_ NPs. The highest crystallite size (~ 15 nm), along with the lowest dislocation value, was observed after using a microwave power of 1000 W (Additional file 1: Fig. S1).

### Dielectric analysis of green-synthesized IONPs

The short-range electrical conduction of a material depends on its dielectric properties. Charged particles in the material experience a displacement as a result of the applied electric field and pile up at the interfaces, resulting in the creation of dipoles. The frequency-dependent dielectric constant of IONPs was obtained using an impedance analyzer with Eq. (6), whereas the tangent loss was calculated using Eq. (7), [38]

6 $$\varepsilon =\left(\complement \mathcal{d}\right)/{\varepsilon }_{o}\mathrm{\rm A}$$

7 $$\mathrm{tan}\delta = 1/\left(2\pi f\varepsilon {\varepsilon }_{o}\rho \right)$$

where ‘C’ represents capacitance, ‘d’ is the specimen’s thickness, ‘A’ is the area, ‘ρ’ is the resistivity, and permittivity of free space is represented by ‘ε_o_’.

**Fig. 3:**
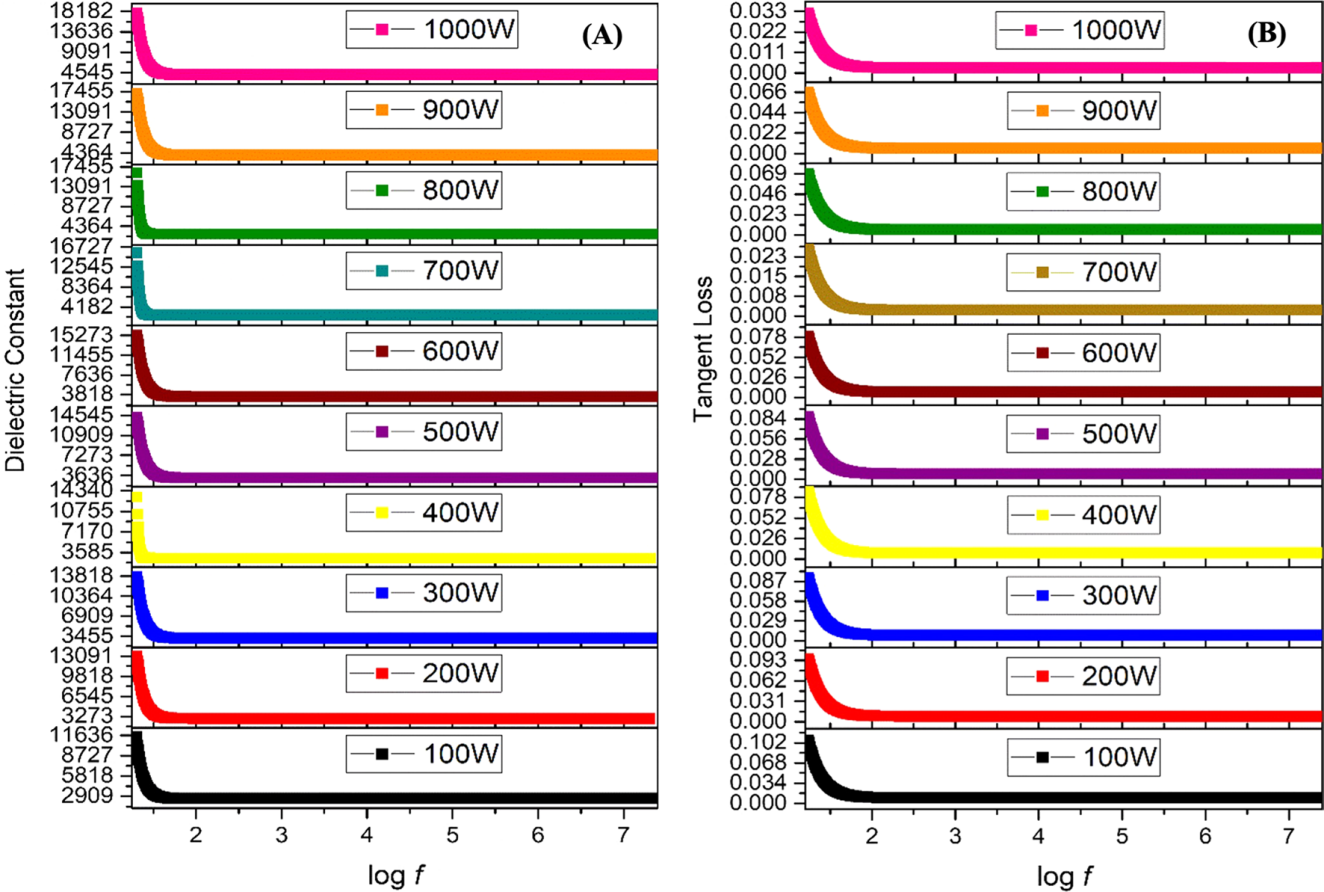
Dielectric analysis of green synthesized IONPs: Room temperature response of dielectric constant (**A**), tangent loss (**B**) at various microwave powers (100–1000 W)

The variation in the dielectric constant and tangent loss as a function of frequency at room temperature are shown in Fig. 3A, B. Different types of polarization, that is, ionic, electronic, orientational, and dipole, are accompanied by a dielectric constant under the effect of an alternating electric field. At low frequencies, charge polarization is dominant, whereas at high frequencies, ionic and electronic polarizations dominate in a polycrystalline material. When an external electric field is applied, dispersion occurs in space charges, and these space charges require some time to align themselves in the direction of an applied field. In the low-frequency space, charges get enough time for their arrangement as per the applied field. However, at high frequencies, charges do not get enough time to orient themselves in the direction of the applied field, thus resulting in low dielectric values (Fig. 3A). The dielectric constant remained almost the same for samples synthesized at low microwave powers, *i.e*., 100–200 W. The small dielectric constant at low microwave power is due to the amorphous behavior of the IONPs, as observed in the XRD patterns (Fig. 2). An increase in the dielectric constant was observed with an increase in microwave power from 300 to 1000 W. The high dielectric constant is attributed to the presence of both Fe^3+^ and Fe^2+^ cations in the Fe_3_O_4_ phase of iron-oxide. Heterogeneity in the Fe_3_O_4_ structure arises because of the existence of Fe^2+^ cations that provide high polarization, leading to a higher dielectric constant [39, 40]. A high dielectric constant can be employed in the agricultural field, that is, in plant pathology, to investigate the activities of pathogenic microbes, including fungi.

The tangent loss of the synthesized NPs is shown in Fig. 3B. The plot of the tangent loss shows normal dispersion behavior, owing to space charge polarization. Relatively, higher values of tangent loss were observed for NPs prepared at low microwave power, i.e., 100–200 W, whereas low values of tangent loss were observed at high microwave powers. Comparison of the dielectric constant and tangent loss for varying microwave powers at log *f* = 5 and log *f* = 1.3 is shown in Additional file 1: Fig. S2.

The conductivity of the synthesized IONPs was calculated using Eq. (8) [38]:

8 $$\sigma =2\pi \mathrm{tan}\delta f\varepsilon {\varepsilon }_{o}$$

Conductivity is categorized into two regions: low-frequency and high-frequency regions. In the low-frequency region, conductivity is known as dc-like conductivity, because of the free charge carriers. However, at the high-frequency region, it is known as ac-like conductivity, because of the bound charge carriers. Additional file 1: Fig. S3A shows the variation in conductivity as a function of frequency for all of the samples prepared using various microwave powers, that is, from 100 to 1000 W. Small values of conductivity, because of the hopping mechanism of charge carriers, are observed even at high frequencies. A comparison of the conductivity values at log *f* = 5 and log *f* = 7.3, using various microwave powers is shown in Additional file 1: Fig. S3B.

Impedance is a property that offers opposition to the flow of electric current. Conduction in nanomaterials can be best comprehended based on complex impedance spectroscopy, which is an effective technique for differentiating the resistive and conducting elements in the circuit. The complex impedance (Z*) was calculated using Eq. (9) [38], while the real (Z') and imaginary (Z'') impedances were calculated using Eqs. (10) and (11), respectively [38]:

9 $${\mathrm{\rm Z}}^{*}={\mathrm{\rm Z}}^{^{\prime}}-j{\mathrm{\rm Z}}^{"}$$

10 $${\mathrm{\rm Z}}^{^{\prime}}=\mathrm{\rm Z}\mathrm{cos}\theta$$

11 $${\mathrm{\rm Z}}^{"}=\mathrm{\rm Z}\mathrm{sin}\theta$$

**Fig. 4:**
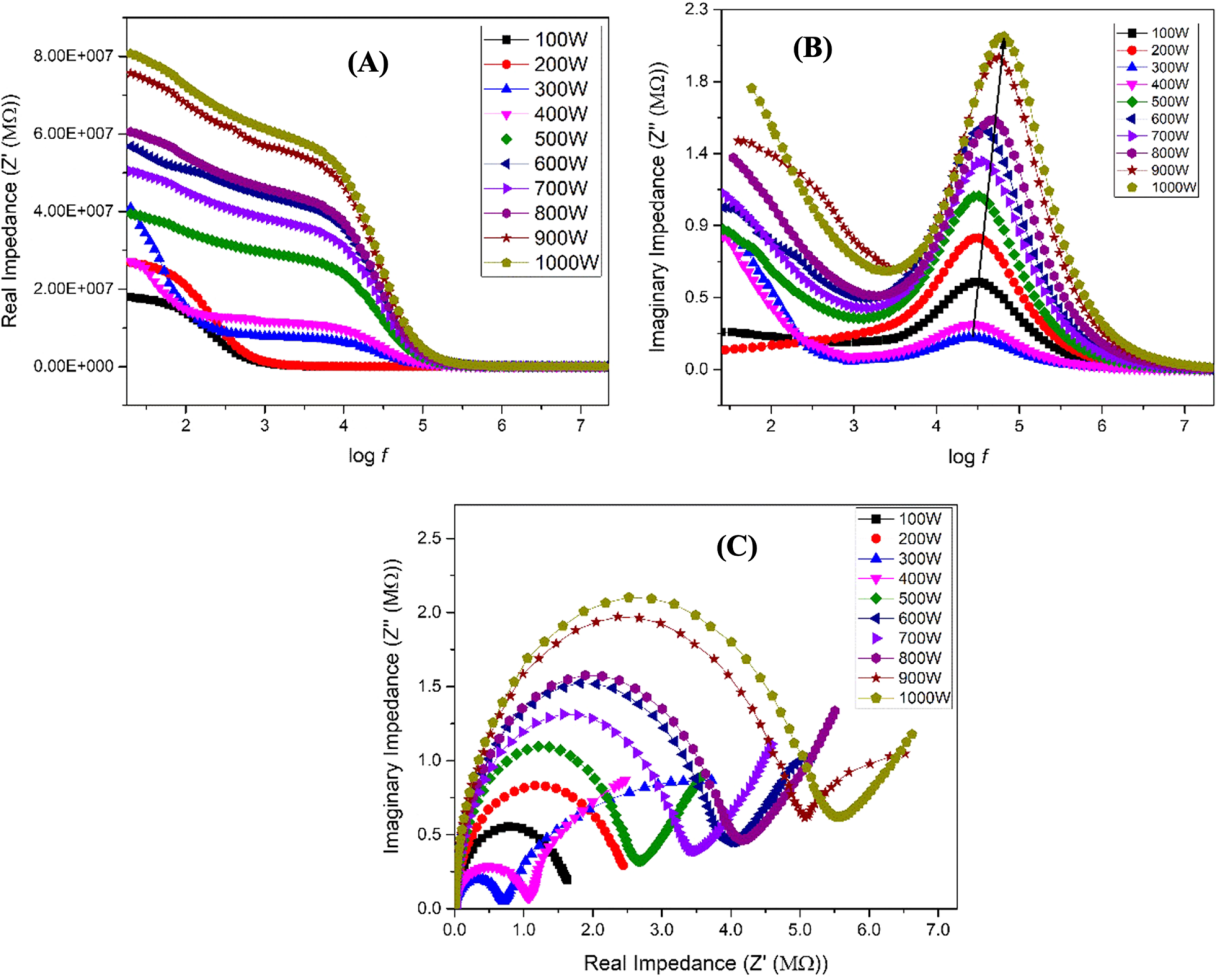
Impedance spectroscopic analysis: **A** Real impedance: **B** Imaginary impedance: **C** and Cole–Cole plots were drawn to extract information about electrical characteristics of green synthesized IONPs

The variation of real and imaginary impedance of IONPs with frequency is depicted in Fig. 4A, B. Two regions are observed in the Z' plots (Fig. 4A). The first is the region of low frequencies, in which there is a slight decrease in Z'. The second is the region in which Z' decreases monotonically and becomes constant at higher frequencies. This behavior is related to the conductivity of charge carriers observed in Fig. 4A, where the conductivity increases at high frequencies [41, 42]. Z'' (Fig. 4B) shows different relaxation peaks at different frequencies for changes in microwave power. Information regarding grains and interface effects in polycrystalline materials can be realized based on these relaxation peaks, which demonstrate different relaxation mechanisms in NPs [42]. The relaxation peaks at different microwave powers, spread across different zones of frequency, explain the distinct participation of grains and grain boundaries. To obtain the exact contribution of grains and grain boundaries to the conduction process, Cole–Cole plots, that is, plots between Z'' and Z', were studied (Fig. 4C). Generally, the Cole–Cole plot contains three semicircles. The first semicircle in the high-frequency regime represents the effect of grain resistance, the second semicircle in the middle frequency range corresponds to the grain boundary resistance, and the third semicircle in the low-frequency range represents the resistance offered by the grain-to-grain boundary interface. These plots provide information on the electrical characteristics of the material (grains and grain boundaries) [43].

### FTIR analysis of green-synthesized IONPs

**Fig. 5:**
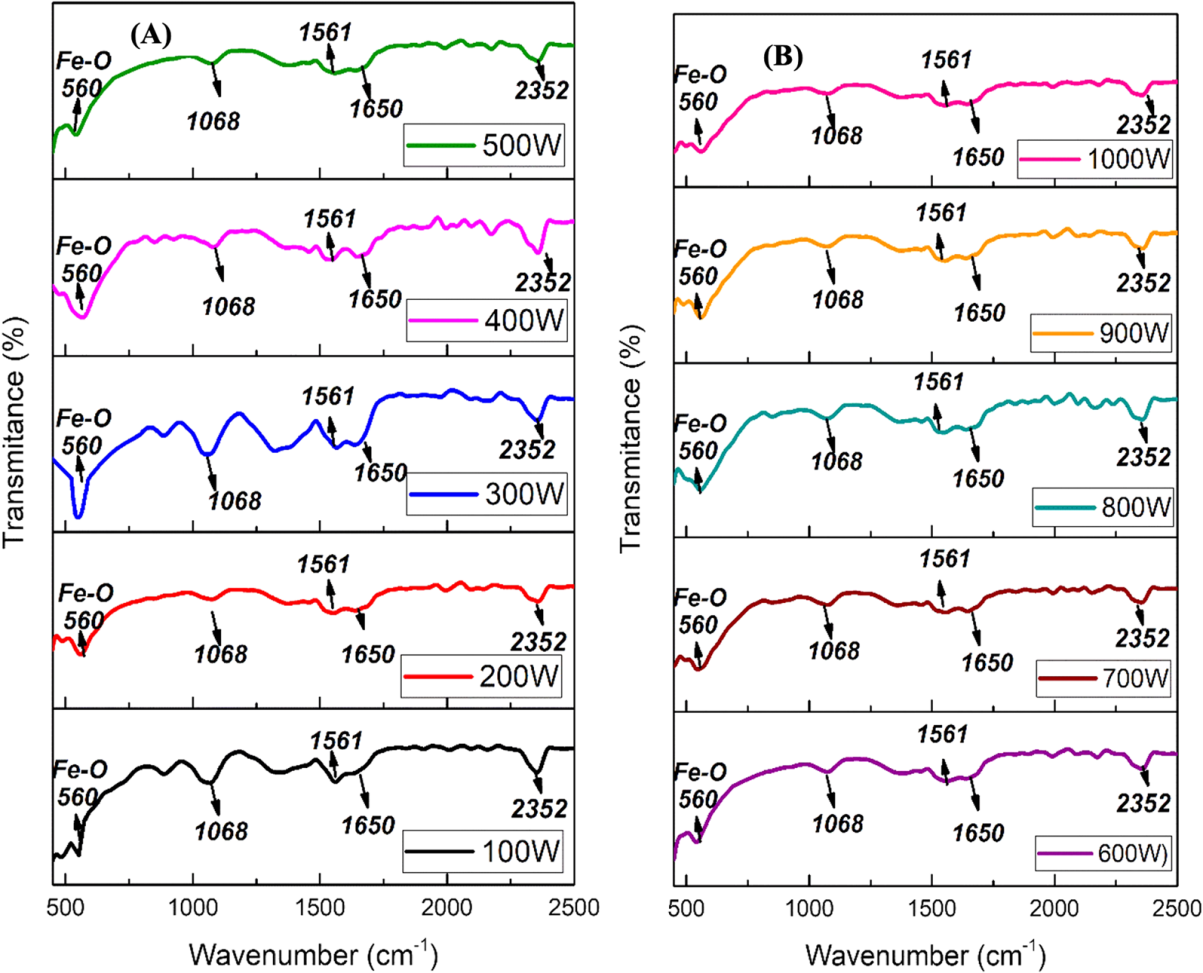
Fourier transform infrared (FTIR) spectroscopic analysis of IONPs: FTIR spectra for green synthesized iron oxide nanoparticles synthesized at various microwave powers by using spinach and black coffee extracts, **A** 100 W- 500 W; **B** 600 W-1000 W in a range of 500–2500 cm^−1^ at 4 cm^−1^ resolutions in diffuse-reflectance mode by using KBr (potassium bromide) pellets in the ratio of 1:100

FTIR spectra of the green-synthesized IONPs using BC extract and dried using various microwave powers are depicted in Fig. 5A, B. The peak appearing at ~ 560 cm^−1^ was assigned to a characteristic band of Fe–O, which is in good agreement with previously reported literature [44]. The band at 1068 cm^−1^ appeared due to C-N stretching. The peaks appearing at 1561 cm^−1^ and 1650 cm^−1^ were ascribed to C = C stretching vibrational bands, due to the presence of aromatic rings/phenolic groups in the BC extract. The presence of phenolic features indicates a capping effect on the surface of IONPs. The absorption band at 2352 cm^−1^ corresponds to atmospheric CO_2_ [45].

### Magnetic response of green-synthesized IONPs

**Fig. 6:**
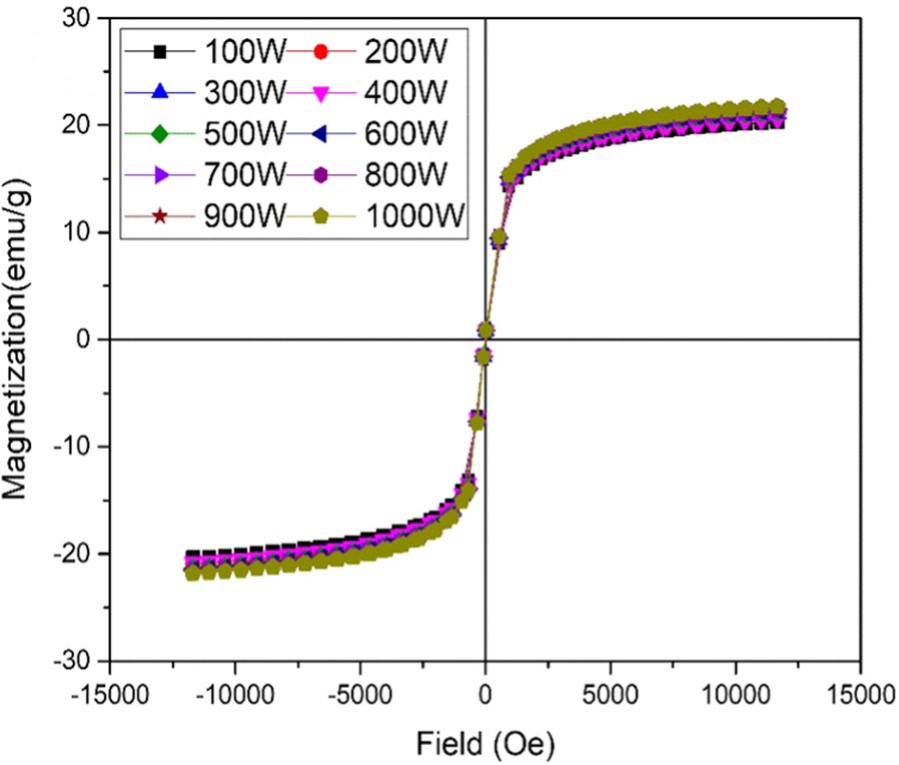
Superparamagnetic response of green synthesized IONPs: Magnetization curves of IONPs obtained by Vibrating sample magnetometer (VSM), synthesized at various microwave powers (100 W- 1000 W). Magnetization unit is represented by emu/g respectively

The super-paramagnetic response of the green-synthesized IONPs at various microwave powers, that is, 100–1000 W, was detected through the M-H loops (Fig. 6). However, high saturation magnetization (~ 21.72 emu/g) was observed for IONPs synthesized at 1000 W. Super-paramagnetic behavior arises when the size of a single domain becomes so small that thermal energy can easily overcome the anisotropy energy barrier. As the particle size decreases, the number of surface spins contributing to the magnetization increases [46]. Such behavior of IONPs makes them potential candidates for agricultural applications in terms of targeted delivery of nutrients and controlled release of pesticides in plants. Variations in saturation magnetization (Ms) were observed using various microwave powers (Additional file 1: Fig. S4).

### X-ray photoelectron spectroscopic (XPS) analysis of green-synthesized IONPs

**Fig. 7:**
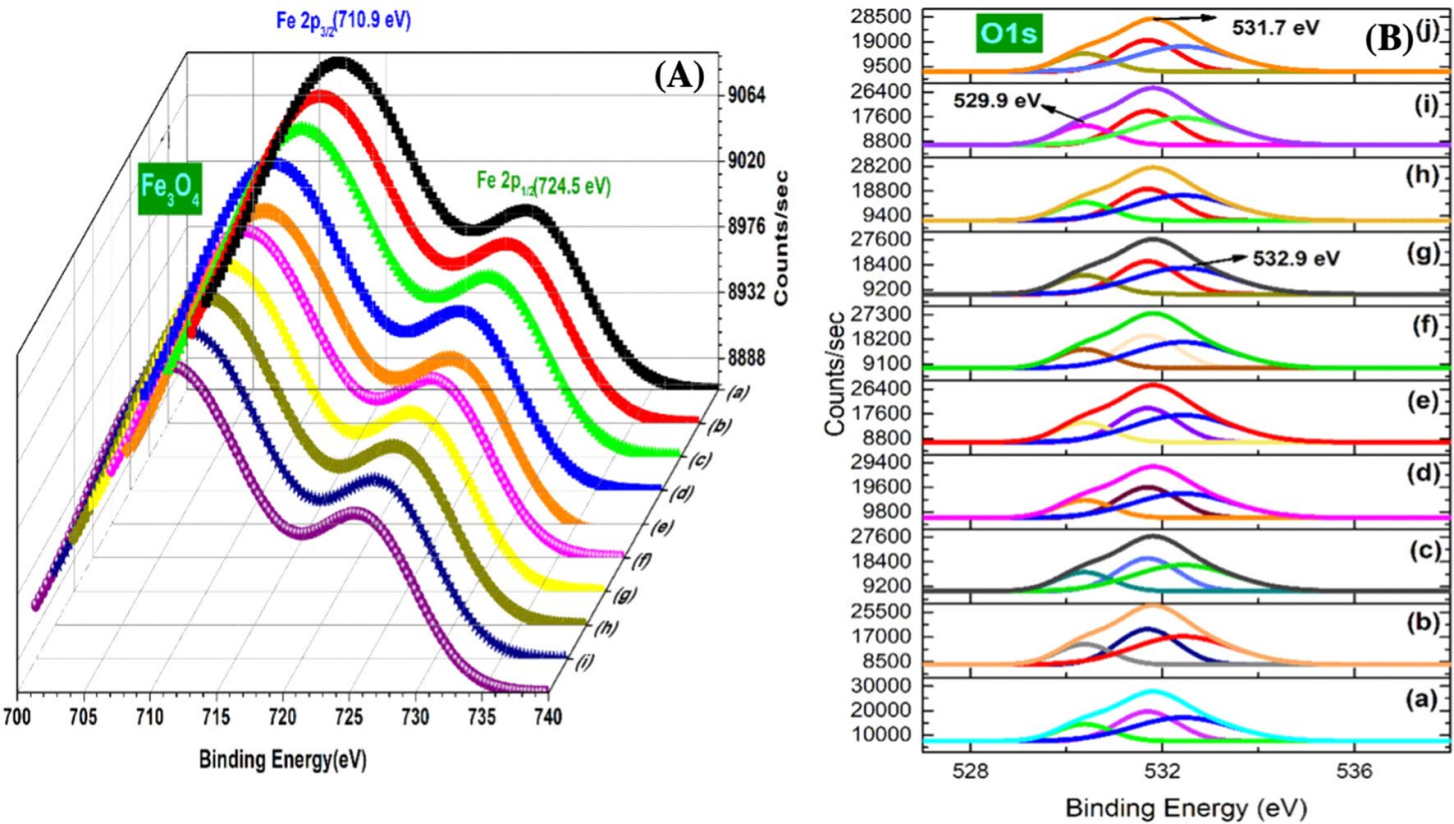
X-ray photoelectron Spectroscopic analysis of IONPs synthesized at various microwave powers (100 W-1000 W: a-i): XPS spectra of (**A**) Fe2p and (**B**) O1s

XPS analysis of IONPs was performed using spinach as a precursor along with BC extract at various microwave powers (100–1000 W), at intervals of 100 W (Fig. 7A, B). It can be observed in Fig. 7A that binding energy peaks of Fe 2p_3/2_ and Fe 2p_1/2_ at 710.9 and 724.5 eV, respectively, are due to magnetite (Fe_3_O_4_) [47]. The split spin–orbit peak of Fe 2p is wide because of the lower chemical shift between Fe^2+^ and Fe^3+^ [48]. Figure 7B represents the spectra of the O1s core level. Peak presence at 529.9 eV is due to the existence of O^−2^ species and that at 531.7 eV is due to the existence of OH^−^ species present on the iron-oxide surface. Another peak at 532.9 eV is associated with adsorbed H_2_O molecules [49].

### Surface morphology and size distribution of green-synthesized IONPs

The benefits of using the microwave-assisted approach, in contrast to chemical methods, include in-core heating of materials, and the specific chemical bonds, which give discerning absorbance, resulting in nano-sized particles with uniform size and shape, as depicted in the microscopic images [SEM and transmission electron microscopy (TEM)]. The morphology and particle size distribution of the green-synthesized IONPs generated at 1000 W power were revealed using SEM and TEM analyses (Additional file 1: Fig. S5). Microscopic images indicated the uniform, spherical to cubical, poly-disperse, less-aggregated, crystalline nature of the obtained IONPs (Additional file 1: Fig. S5A and C), which confirms the purity of the sample without the presence of any other phases. Additional file 1: Fig. S5B shows the particle size distribution of IONPs, as determined using SEM, which was in the range of 3–23 nm, with an average diameter of 4.99 ± 0.17 nm. Correspondingly, the mean particle size of 4.08 ± 0.19 nm was determined using TEM, varying from 1 to 15 nm, conferring to Gaussian fit of the particle size distribution (Additional file 1: Fig. S5D). These results displayed the narrow size distribution of green-synthesized IONPs, consistent with previous literature [50–53].

### IONPs as an inducer of anti-fungal activity, leading to inhibition of fungal growth and spore germination

**Fig. 8:**
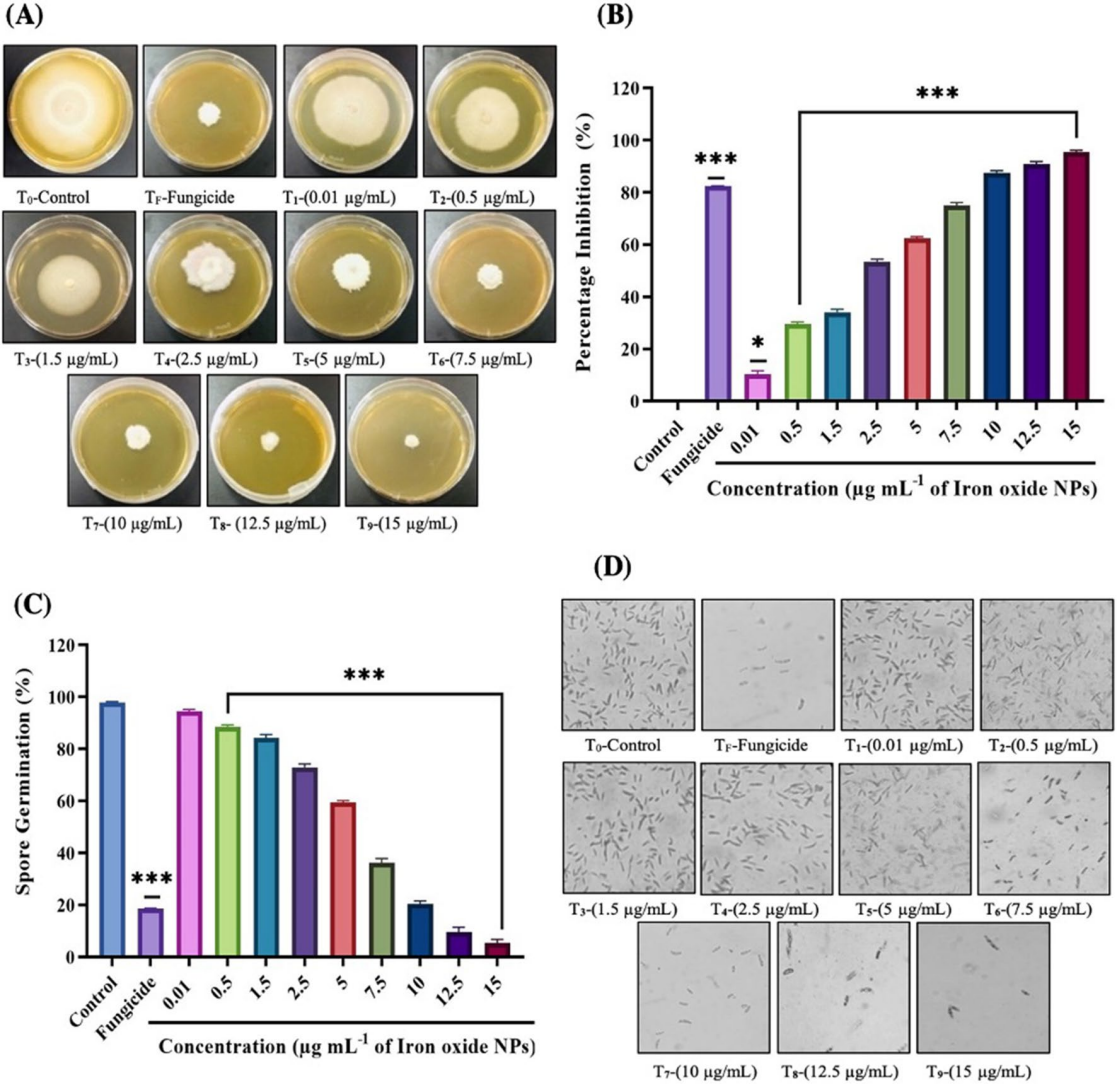
Comparing the effect of Iron-oxide nanoparticles (IONPs) on *F. oxysporum* mycelial radial-growth and spore-germination at various concentrations (0.01–15 µg/mL) in parallel to the control and fungicide treatment after seven days of post-incubation at 28 °C. **A** Plates indicating antifungal activity of IONPs in PDA plates, **B** Showing percentage inhibition graph of IONPs on mycelial growth of *F. oxysporum,* **C** Represents graph of percentage spore’s germination and **D** Indicating fungal spores’ germination rate treated with different concentrations of NPs viewed under the light microscope (10 × magnification). Data represents a mean ± SD (n = 3) of three replicates indicating significant-difference (**p < 0.01; *** p < 0.001) as compared to control by one-way-ANOVA (P < 0.05) and Tukey’s-multiple comparing tests

We investigated the in vitro anti-fungal activity of IONPs synthesized at various microwave powers (100–1000 W), as shown in Additional file 1: Table S1. After initial screening, the IONPs at 1000 W were selected and used for the next experiments, which also justified the phenomena of the highest magnetic saturation at this microwave power. The emergence of resistance in plant fungal pathogens against agro-chemicals has led to the development of more efficient eco-friendly antifungal agents. Therefore, the anti-fungal potential of NPs is beneficial in the agriculture sector, as these have now emerged as “innovative-generation fungicides” [54]. Figure 8 shows the effect of IONPs on fungal mycelial growth and spore germination at various concentrations (0.01–15 µg/mL) against *F. oxysporum,* the causal agent of tomato wilt.

IONPs exhibited a strong inhibitory effect on the radial growth of fungal mycelia on PDA medium, as compared to the control treatment (Fig. 8A). It is evident from the results that the anti-fungal activity of IONPs significantly increased in a dose-dependent manner. Seven days post-inoculation, 15 µg/mL of IONPs strikingly minimized *F. oxysporum* growth by 95.45 ± 0.41%, in contrast to the control treatment. The lower concentrations, ranging 0.01–1.5 µg/mL, displayed minimum growth inhibition, *i.e.*, below 50%, whereas, fungicide exposure yielded 89.77 ± 1.24%, relative to the growth rate in the control group (Fig. 8B). Previous studies have indicated the anti-microbial activity of IONPs, and their findings suggest that this activity increases gradually from lower to higher concentrations [22, 23, 55].

The sporicidal activity of IONPs on the spore germination of *F. oxysporum* is illustrated in Fig. 8C, D. Disruption of fungal membrane indicates that the inhibiting action of NPs, as observed in this study, is due to the biocidal action of NPs, which retain a large surface area and are readily attached and absorbed to disassemble the microbial cell membrane, leading to deterioration of intracellular organelles, eventually resulting in the death of microorganisms [56, 57]. After six hours of incubation with various concentrations of IONPs, the microscopic images revealed that the spore suspension of *F. oxysporum* displayed a sharp decline in spore germination rate, in comparison to the untreated control samples (Fig. 8D). In the fungal life cycle, spore germination and maturation are vital phases for successful plant colonization, but once germination is subdued after treatment with metal oxide NPs, the spores cannot develop into mature mycelium, to initiate the infection cycle [35]. The results indicated that the germination rate of spores gradually decreased in response to increasing concentrations. A statistically significant reduction in germination rate was observed at concentrations ranging between 1.5 and 15 µg/mL, while the minimum germination rate was up to 5.38 ± 1.38% upon treatment with 15 µg/mL IONP, in comparison to that in the control group (97.2 ± 1.13%). Analogously, in the case of fungicide, significant inhibition of spore germination was found (18.56 ± 0.86%) (Fig. 8C). Devi et al*.* worked on two fungal species (*A. niger* and *M. piriformis*) and proposed that the greater surface interaction between the IONPs and fungal membranes played a significant role in their anti-fungal activity [58]. Similarly, Saleem et al*.* also demonstrated the anti-fungal potency of green-synthesized IONPs against *A. flavus* and *F. oxysporum*, suggesting that IONPs have the potential to be used for biological applications [59].

### IONPs induce changes in cell wall morphology, viability, and ROS production in *F. oxysporum*

**Fig. 9:**
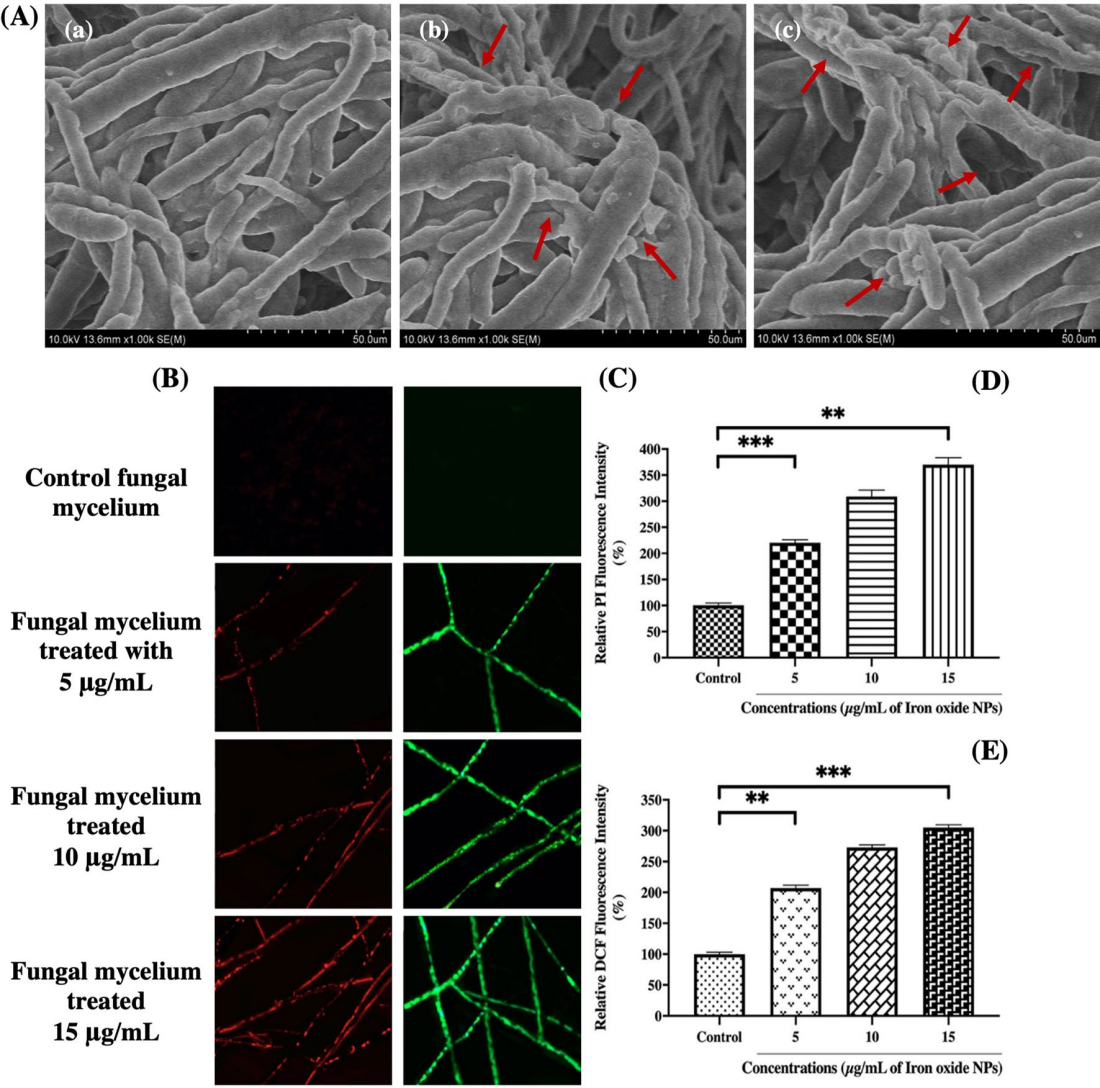
Assessing the effect of Iron-oxide nanoparticles on fungal morphology, intracellular reactive oxygen species (ROS) and membrane integrity. **A** Scanning-electron-microscope (SEM) micrographs of *F. oxysporum* hyphae treated with IONPs **a** sterilized-water (control), **b** 10 µg/mL, **c** 15 µg/mL; **B**, **C** showing red (RFP) and green (GFP) fluorescent protein micrographs of control and IONPs treated fungal hyphae, Scale bar: 10 µm, **D**, **E** representing fluorescent intensity of propidium iodide (PI) and dichloro-dihydro-fluorescein diacetate (DCFH-DA) in dose-dependent manner. Graph denotes significant-difference (**P < 0.01; *** P < 0.001) in fluorescence-intensity between different concentrations of nanoparticle and control group performed by one-way-ANOVA at P < 0.05 and Tukey multiple comparisons analysis. Each bar represents a mean ± SD of three independent experiments

The SEM micrographs revealed malformed mycelia after treatment with IONPs, which can be attributed to distortion of chitin synthesis and cell envelope, which shields the leakage of cellular components into the extracellular environment [19]. SEM visualization indicated the detrimental effects of IONPs on *F. oxysporum*. The IONP-treated mycelia showed some eccentric morphological characteristics, as compared to the control (Fig. 9A). In the control, cylindrical-shaped mycelia had a healthy smooth turgid surface with clear conidiation (Fig. 9A-a). However, upon treatment with IONPs, remarkable structural changes were induced in the fungal hyphae, as observed in Fig. 9A-b and c. Upon treatment with 10 µg/mL IONP, hyphae became deformed, showing irregular shrinkage with minute granules on the surface (Fig. 9A-b). The impairment was intensified upon treatment with 15 µg/mL IONP, and the hyphae became recessed, slender, and stacked together, including rifts or blebs (Fig. 9A-c). Earlier investigations suggested that internalization of magnetic NPs through microbial membranes causes toxicity, such as the discharge of metal ions, affecting protein synchronization and cellular homeostasis, lipid peroxidation and nucleic acid impairment by the accumulation of ROS and mutilation of cell integrity by means of membrane depolarization [60].

The anti-fungal properties of IONPs were further verified using fluorescent dyes, such as PI and H_2_DCFDA. The fluorescence intensity of fungal hyphae was significantly enhanced in a dose-dependent manner upon treatment with IONPs, in contrast to that in the untreated control. A similar outcome has been observed for IONPs bound with amphotericin B, by means of a reaction between the aldehyde and amine groups against Candida strains [61]. PI is a DNA-fluorescent probe that invades the disrupted plasma membrane of a cell, subsequently emitting red fluorescence from the stained nucleus [62]. The effect of IONPs on the membrane integrity of *F. oxysporum* in the control and treated fungal mycelial samples are depicted in Fig. 9B. As shown in the red fluorescence protein-containing images, a slight red fluorescence was detected in the control hyphae, whereas stronger fluorescence was observed in the treated ones. After 15 min of exposure, the PI fluorescence intensities in the treated groups (with 5, 10, and 15 µg/mL IONP) were 2.21-, 3.09-, and 3.69-fold higher, respectively, than those in the control group (Fig. 9D). The green fluorescence protein-containing micrographs of the control and IONP-treated fungal mycelia, depicting ROS accumulation, are illustrated in Fig. 9C. Treatment of *F. oxysporum* with 5, 10, and 15 µg/mL IONPs significantly increased H_2_DCFDA fluorescence intensity by 2.07-, 2.73-, and 3.05-fold, respectively, as compared to that in the control (Fig. 9E). Cell genotoxicity and cytotoxicity are affected by the surface charge of IONPs, and NPs with positive charges tend to be more lethal, endure adsorptive endocytosis, and display non-specific interactions with the negatively charged cell membrane, hence distressing the membrane permeability by enhancing intracellular accumulation [63]. Thus far, it has been documented that IONPs exert anti-microbial effects by inducing the production of ROS, following disruption of electron transport by microbes, NADH oxidation, and cellular homeostasis, thus contributing to anti-fungal effects [64]. No doubt, magnetic oxide NPs retain the ability to aggravate oxidative stress by disturbing the redox potential of cells and augmenting the host anti-microbial resistance, by targeting the infection sites with a direct approach, to eradicate microbial pathogens [61–65].

Our results indicate that IONPs inactivate the oxidation–reduction balance by generating ROS, which may be associated with the mechanism of action of magnetic NPs, such as pore formation in the cell membrane, and stipulating the transport of NPs into Fusarium cells. Earlier reports revealed that after uptake of IONPs, the cells produce ROS via either pathway: Iron ions contribute to the Haber–Weiss cycle by releasing ions into the cytosol, where chelation by adenosine-phosphate or citrate occurs; or the surface of IONPs catalyzes Fenton reaction or Haber–Weiss cycle, which could have a detrimental effect on fungal cells; highly reactive hydroxyl radicals are formed as a result of both pathways [66]. Comparing the fluorescence intensities, as shown above, indicates that the fungal-nano interactions were relatively stronger upon treatment with IONPs, which eventually increased the variation in free-energy content, resulting in increased ROS generation. In line with the current investigation, Arakha et al*.* found that chitosan-coated IONPs in culture media can enhance ROS production by altering the interaction pattern among bio-nano interfaces, thus playing a critical role in the anti-microbial affinity of IONPs [27].

### IONPs as an inducer of DNA fragmentation in *F. oxysporum*

DNA fragmentation is another key biochemical feature of apoptosis (programmed cell death) [67]. The inter-nucleosomal cleavage of genomic DNA was studied in *F. oxysporum* subjected to treatment with various concentrations of IONPs. DNA was isolated from fungal cells and analyzed by means of agarose gel electrophoresis. The electrophoretogram is shown in Fig. S6. As can be seen in the gel image, a typical DNA band was formed in the control group, while “DNA-laddering” of non-chronological DNA fragments was found in the groups treated with IONPs. Moreover, it was observed that DNA-cleavage increased in IONP-treated samples in a concentration-dependent manner. A gel containing DNA of *F. oxysporum* also indicated single high molecular DNA bands in lanes 1, 2, and 3, treated with 0.01, 0.5 and 1.5 µg/mL IONPs, respectively. However, the intensities of these DNA bands were lower than those in the control group (lane 10). Additionally, the smeared DNA in all lanes was less than 1 kb and appeared weaker than the control. Smeared-DNA almost disappeared in lanes 8 and 9, implying that IONPs completely impaired the fungal DNA. The results indicated that the anti-fungal effect of IONPs on fungal cells was triggered by the initiation of cell apoptosis. Alarifi et al*.* observed the breakage of DNA double helix strands by IONPs in a time- and dose-dependent manner [68]. ROS has been implicated in DNA mutilation by IONPs, affecting DNA bases such as pyrimidine and purine and contributing to reduced biofilm formation in bacterial cells [29].

### Impact of IONPs on tomato growth parameters

**Fig. 10:**
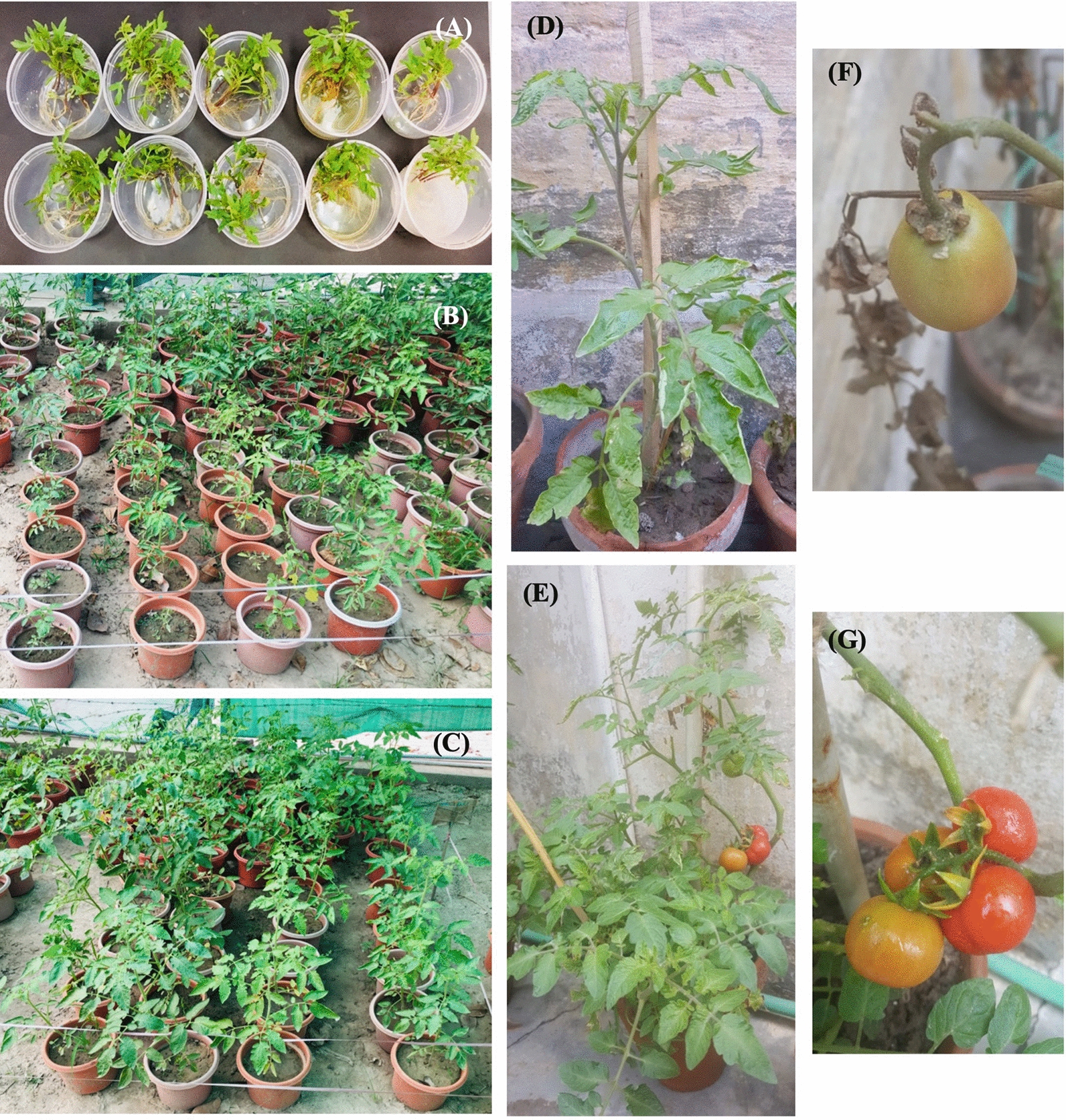
Application of IONPs on infected tomatoes under greenhouse conditions. **A** Showing root dipping protocol-tomato seedlings treated with various concentrations of IONPs for two hours at room temperature, **B**, **C** Representative pictures of control and treated (nanoparticles concentrations) tomato plants under green-house conditions arranged with replicates, **D** Representing control tomato plant (inoculated with *F. oxysporum* only), **E** illustrating treated tomato plant with the best concentration of IONPs (12.5 µg/mL) and **F**, **G** Tomato fruits of control (upper) and treatment (lower) group

All concentrations of IONPs (0.01–15 µg/mL) significantly enhanced the growth parameters (root and shoot length), biomass (fresh and dry weight), and plant height of tomato plants infected with Fusarium wilt in the pot bioassay (Figs. 10 and Additional file 1: Fig. S7). Root, shoot length, and germination rate are the main indicators used to investigate the impact of NPs in different plant species [69]. The IONP concentration of 12.5 µg/mL presented the best results, by inducing a substantial increase in growth attributes, in comparison to the control and other treatments. A gradual increase in plant height was observed for all treatments; however, plants treated with 10, 12.5, and 15 µg/mL of IONPs showed maximum plant heights of 45.1, 48.9, and 43 cm, respectively (Additional file 1: Fig. S7A), exceeding the control treatment by 64, 77.8, and 56.3%, respectively. Similarly, a consistent result was observed upon fungicide treatment, with an increase of 51.6%. Correspondingly, the root and shoot lengths of plants were substantially improved after treatment with IONPs (Additional file 1: Fig. S7B). Furthermore, the average root and shoot lengths upon treatment with IONP concentrations 5–15 µg/mL were greatly analogous to the control treatment; predominantly, treatment with IONP at a concentration of 12.5 µg/mL showed an increase of 76.3% (roots) and 79.05% (shoots). Moreover, a statistically significant difference was found in the plant biomass (fresh and dry weight) upon IONP treatment, in contrast to the control treatment (Additional file 1: Fig. S7C). The fresh and dry weights were superior upon treatment with 12.5 µg/mL IONP, surpassing the control by 60.9 and 67.1%, respectively. Fungicide treatments followed the same trend for fresh and dry weight, with an increase of 43.8% and 47.7%, respectively, from the control. Earlier studies have reported an increase in seed germination, plant biomass, seedling growth, and yield after the application of IONPs [70–74]. Treatment of tomato seeds with Fe_3_O_4_ NPs has no side effects on plant growth and development [75]. Similarly, our results verified the previous research by observing the enhancement in growth parameters of tomato plants treated with various concentrations of IONPs.

### Impact of IONPs on disease attributes of tomato wilt

The virulence of many pathogens relies on iron procurement, and microbial infections can be avoided by using iron-chelating products that inhibit the pathogen’s ability to approach iron [76]. To consider IONPs as an agricultural anti-fungal agent, pot bioassays were performed to evaluate the potential of NPs in inhibiting Fusarium wilt infection under greenhouse conditions (Fig. 10D, E). As shown in Additional file 1: Fig. S8A, B, treatment of infected tomato seedlings with IONPs reduced the severity and incidence of Fusarium wilt caused by *F. oxysporum*. Furthermore, a clear positive correlation exists between NP concentrations and disease index. The disease severity in control plants was specifically severe 25 days post-inoculation and reached 96.67%; however, the disease severity of tomato seedlings exposed to IONPs at concentrations of 10, 12.5, and 15 µg/mL reduced to 43.33, 47.78, and 45%, respectively. Upon fungicide treatment, a decline of 55% was achieved in disease severity (Additional file 1: Fig. S8A). The corresponding disease incidence in IONP-treated plants was 46.6, 33.3, and 48.7% at the IONP doses of 10, 12.5, and 15 µg/mL, respectively, in contrast to 100% in the control plants (Additional file 1: Fig. S8B). Plants activate a toxic oxidative burst by increasing iron levels, to minimize pathogen virulence; root mutualistic interactions also encounter phyto-diseases via iron uptake, and competition for iron acquisition induces a systemic resistance that signals components in roots for iron uptake [77]. To date, there have been no studies on the application of IONPs to combat plant diseases under field conditions. Thus, the present study could be considered novel, as these results indicate that NPs have the potential to become a part of disease management.

### Impact of IONPs on photosynthetic pigments

The effects of IONPs on the photosynthetic pigments were also assessed in this study by comparing diseased plants with treated ones (Additional file 1: Fig. S9A, B). Plant stress can also be indicated by changes in photosynthetic pigments [78]. The results indicated that various treatments with IONPs increased the photosynthetic pigments. After exposure to 12.5 µg/mL IONPs, total chlorophyll and carotenoid contents in the treated plants were significantly increased by 75.6 and 70.3%, respectively, in comparison to the control; however, lower doses (0.01 and 0.5 µg/mL) showed a decline of up to 27.7 and 1.94%, respectively, for chlorophyll content and 19.05 and 8.54%, respectively, for carotenoid content. Askary et al*.* reported similar results with the application of nano-iron fertilizers [9]. Compared to the control, increased pigment production was detected upon fungicide treatment, with elevations of 56.1 and 55.1% (p < 0.001), respectively. Iron plays a vital role in chlorophyll synthesis. Iron chlorosis reduces the level of photosynthetic pigments in plants, thus affecting the process of photosynthesis and is more frequent in photosystem II, analogous to photosystem I [9]. The results demonstrated that higher doses of IONPs enhanced the synthesis of photosynthetic pigments in diseased plants by reducing chlorosis.

### Impact of IONPs on phenolic content and anti-oxidant enzymatic activities

**Fig. 11:**
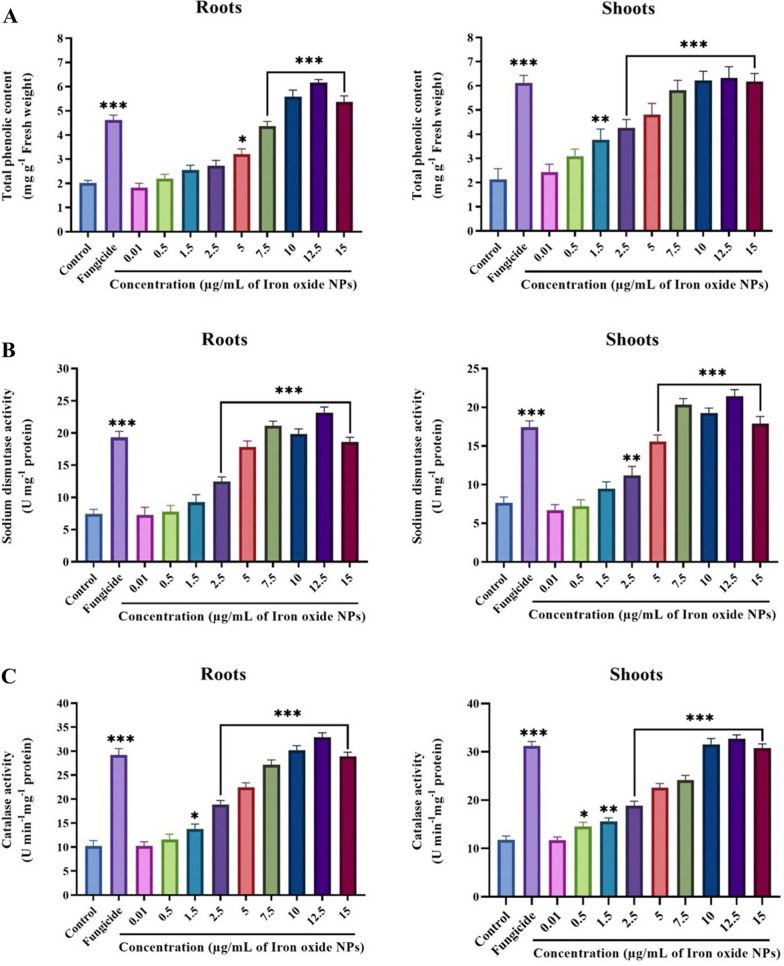
Effect of various concentrations of IONPs on phenolic and antioxidant enzymes: Phenolic content (**A**), SOD (**B**) and CAT (**C**) in the roots and shoots of tomato plants infected with *Fusarium oxysporum* under pot condition. Significant-difference (* P < 0.05; **P < 0.01; *** P < 0.001) among different concentrations of IONPs and control group performed by one-way-ANOVA at P < 0.05 and Tukey multiple comparisons analysis. Error bar represents a mean ± SD of five replicates

**Fig.12:**
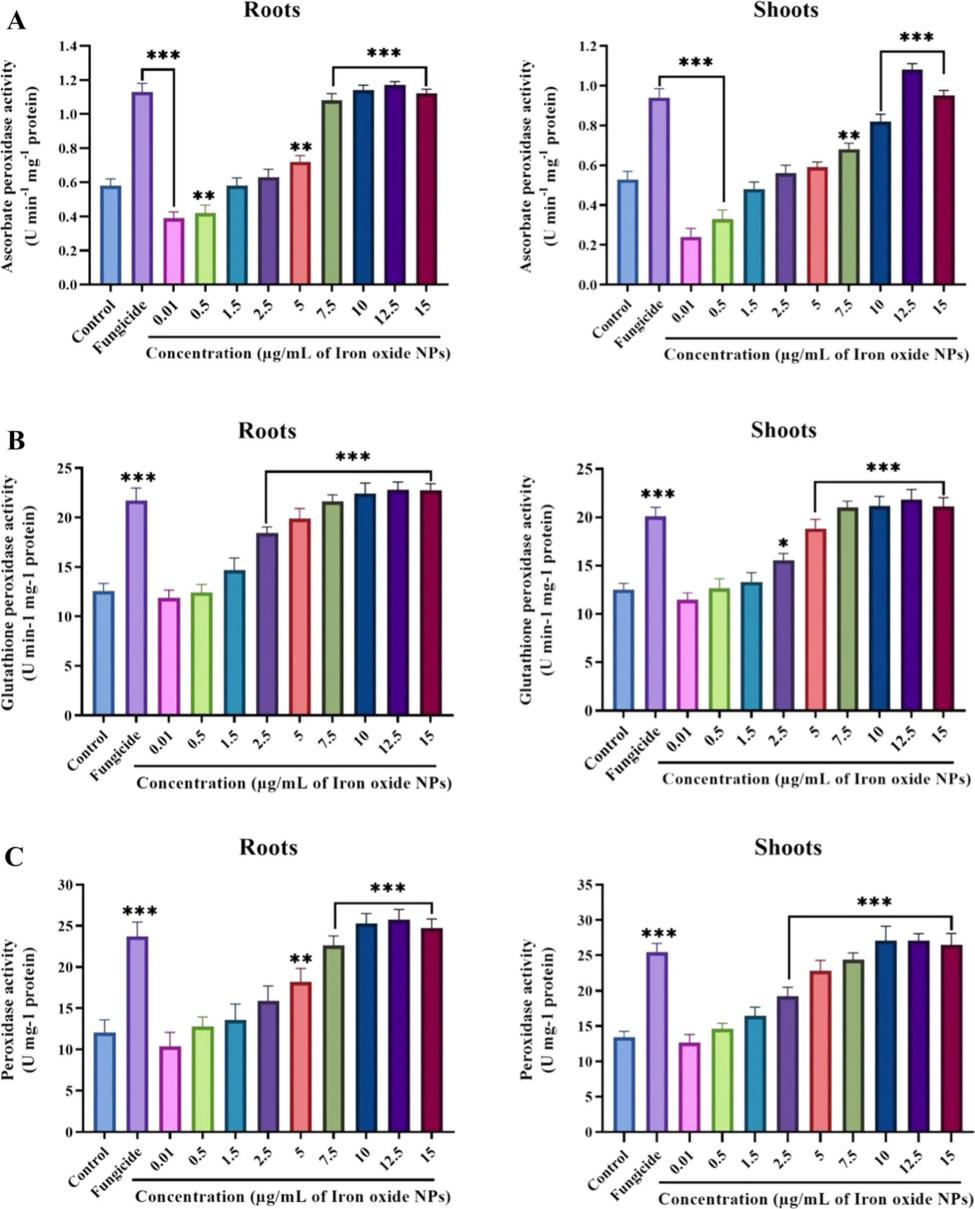
Effect of various concentrations of IONPs on antioxidant defense enzymes: APX (**A**), GPX (**B**) and POD (**C**) in the roots and shoots of tomato plants infected with *Fusarium oxysporum* under pot condition. Significant-difference (*P < 0.05; **P < 0.01; ***P < 0.001) among different concentrations of IONPs and control group performed by one-way-ANOVA at P < 0.05 and Tukey multiple comparisons analysis. Error bar represents a mean ± SD of five replicates.

Plants endure a stressful environment by generating higher quantities of anti-oxidant enzymes, which enhances tolerance against oxidative bursts [13]. Figures 11, 12 and Additional file 1: Fig. S10 shows the phenolic content and activities of anti-oxidant enzymes (SOD, CAT, APX, GPX, and POD) in the roots and shoots of diseased tomato plants, in the presence of 0.01–15 µg/mL IONPs. Defensive responses in plants induced as an outcome of biotic-stress commonly involve phenolic compounds [79]. The total phenolic content in the roots and shoots of tomato plants significantly increased with increasing concentrations of IONPs (Fig. 11A). In comparison to the control, the total phenolic content in plants treated with 12.5 µg/mL of IONPs showed an increase of 3.04-and 2.96-fold in the roots and shoots, respectively. Upon fungicide treatment, the total phenolic content in the roots and shoots increased by up to 2.28- and 2.87-fold, respectively. Avio et al. reported that increased phenolic content in lettuce infected with *Rhizoglomus irregulare* was positively associated with anti-oxidant enzyme activities [80]. Increased phenolic compounds were also detected in the Molvadian balm under salinity stress, upon application of IONPs [81]. Nourozi et al*.* demonstrated that IONPs act as an abiotic elicitor in *Dracocephalum kotschyi*, thus enhancing the accumulation of phenolic compounds [82].

SOD, a metalloenzyme, plays a key role in the plant defense system by mobilizing the disproportionation of free hydroxyl radicals (O_2_^•–^) to H_2_O_2_, to mediate ROS toxicity [83]. SOD activity was significantly enhanced in the roots and shoots of infected tomato plants exposed to IONPs, in parallel to the control treatment. The maximum activity, both in roots and shoots was recorded at the IONP dose of 12.5 µg/mL, which was 3.11- and 2.79-fold higher, respectively, than the control treatment, while it dropped to 0.39-fold and 0.42-fold, respectively, at the IONP dose of 15 µg/mL (Fig. 11B). Rui et al*.* observed increased SOD activity in peanut plants after treatment with IONPs, as compared to the control and Fe-EDTA treatments [84]. Interaction of IONPs and stress environment (salinity) enhances the activity of SOD enzyme, since iron is responsible for higher production of SOD, which leads to oxidative stress [82]; subsequently, the scavenging activity of some other anti-oxidant enzymes such as POD, APX, and CAT can possibly reduce the level of H_2_O_2_, leading to improved plant immunity against the ROS burst [85]. Hence, lower SOD activity at the highest concentration of IONPs may induce a reduction in ROS scavenging, which ultimately increases injury to the plants [86].

In plants, cellular destruction is prevented by CAT, which is involved in regulating H_2_O_2_ levels in tissues, by causing its disintegration into oxygen and water molecules [87]. CAT activity presented a dose-dependent effect, with a higher concentration of IONPs displaying greater enzymatic activity in both the roots and shoots (Fig. 11C). The CAT activity in tomato plants treated with various concentrations of IONPs increased by 1–3.21-fold in the roots and by 1.2–2.7-fold in the shoots, as compared to the control treatment (p < 0.05). Additionally, it can be assumed that higher CAT activity is interrelated with greater production of H_2_O_2_ and SOD activities [13]. APX consumes ascorbate, and GPX employs glutathione as an electron donor, to downgrade ROS levels [88]. The activities of APX and GPX were significantly enhanced after the application of IONPs to both the roots and shoots. In plants treated with 12.5 µg/mL of IONPs, both roots and shoots displayed APX activities that were 1.98- and 2.03-fold higher, respectively, than that in the control (Fig. 12A). A similar trend was observed for GPX activity, which gradually increased with doses of IONPs, surpassing the control by 71.8 and 73.7% at an IONP dose of 12.5 µg/mL in the roots and shoots, respectively (Fig. 12B). Both APX and GPX activities decreased at the IONP dose of 15 µg/mL. Peroxidase is another defense-related enzyme that utilizes pyrogallol and guaiacol for detoxification of H_2_O_2_ and is involved in activating plant resistance against invading pathogens and wound-healing [87]. A significant elevation was observed in POD activity in the roots and shoots, upon exposure to IONPs. In comparison to the control, peroxidase activity was enhanced by 2.09-, 2.13-, and 2.05-fold in the roots and by 2-, 2.02-, and 1.9-fold in the shoots, at 10, 12.5, and 15 µg/mL IONP, respectively (Fig. 12C). However, in case of all enzymatic activities, fungicide treatment also resulted in a significant increase in comparison to the control treatment. Enzymes such as CAT, APX, and POD containing iron groups participate in plant metabolism by neutralizing hydrogen peroxide [89]. In agreement with the current work, earlier studies also reported an increase in secondary metabolites and anti-oxidant enzymatic activities after treatment with IONPs, such as the *Citrus-maxima* plant [90], *Hyoscyamus reticulatus* [91], *Dracocephalum kotschyi* [82], *Oenothera biennis* [92], and *Dracocephalum moldavica* [81].

### Impact of IONPs on growth and non-enzymatic compounds of tomato fruit

Fruit quality is imperative for merchandise. Pulpy fruits are putrescible, and various biotic and abiotic agents can deteriorate the quality of the product [93]. Table 1 indicates substantial improvement in fruit variables (average weight and number) and non-enzymatic compounds of tomato fruit exposed to various concentrations of IONPs (Fig. 10F, G). The average fruit weight increased significantly (by 48.8%) in the presence of 12.5 µg/mL IONPs, in comparison to that in the control. The same trend was noted for fungicide treatment, which surpassed the control by 46.2%. Similarly, at the same concentration (12.5 µg/mL), the highest number of fruits (32.6 per plant) was obtained, which was 51.6% higher than that in the control. Kumar et al*.* reported an increase in fruit mass and fruit number per plant in strawberry, after the combined application of iron-oxide and zinc-oxide NPs [94]. Furthermore, Hernandez-Hernandez et al*.* observed an increase of 25% in tomato fruit weight after the application of selenium and copper NPs [95].

**Table 1:** Average weight, number, antioxidant compounds and protein content in tomato fruits treated with IONPs

Treatments	Average fruit weight (g)	Fruits number	Lycopene (mg 100 g^−1^ FW)	Flavonoids (mg 100 g^−1^ FW)	Vitamin C (mg 100 g^−1^ FW)	Protein (Umin^−1^ mg^−1^)
Control	59.61	21.53	2.16	14.12	15.82	5.26
Fungicide	87.19^***^	30.12^***^	3.42^**^	20.42^***^	17.42^*^	8.92^***^
0.01 µg/mL-IONPs	37.71^***^	16.89^***^	1.84	13.92	14.29^*^	4.75
0.5 µg/mL-IONPs	42.29^***^	18.67^***^	1.98	14.19	14.75	4.86
1.5 µg/mL-IONPs	57.81	20.53	2.06	15.25	15.37	5.12
2.5 µg/mL-IONPs	69.63^***^	21.84	2.49	16.52^***^	16.21	5.67
5 µg/mL-IONPs	74.64^***^	23.74^***^	2.87	18.83^***^	16.64	6.74^**^
7.5 µg/mL-IONPs	79.93^***^	25.52^***^	3.15	19.42^***^	17.07	8.49^***^
10 µg/mL-IONPs	83.59^***^	28.59^***^	3.66^***^	20.76^***^	17.86^***^	8.74^***^
12.5 µg/mL-IONPs	88.75^***^	32.63^***^	3.95^***^	21.37^***^	18.12^***^	9.15^***^
15 µg/mL-IONPs	84.26^***^	31.09^***^	3.78^***^	21.03^***^	18.02^***^	8.96^***^

Mean values of five replicates are presented for each treatment. Asterisks represent (*p < 0.05, **p < 0.01, and ***p < 0.001) significant differences as governed by ANOVA

Tomato fruit contains carotenoids, such as lycopene, which is a potent anti-oxidant that counteracts ROS. Another function of lycopene in plants is associated with chemo- and photoprotection [28, 96, 97]. The lycopene content of tomato fruit significantly increased at 10, 12.5, and 15 µg/mL of IONPs, with an increase of 69.4, 82.8, and 75%, respectively, in contrast to the control. Treatment with lower doses (0.01–1.5 µg/mL) showed non-significant decreases of 14.8, 8.33 and 4.62%, respectively, as compared to the control. Previous studies have reported that soil and foliar treatment of TiO_2_, ZnO, and CuO NPs enhanced the lycopene content in tomato plants [98, 99]. Foliar application of various treatments of copper NPs significantly increased the lycopene content in tomatoes from 56.8 to 105.3%, in comparison to the control [100].

Flavonoids are naturally available phytochemicals present in vegetables and fruits that have anti-cancer properties and act as anti-oxidants, to regulate ROS homeostasis [101]. The flavonoid content in tomato fruits was significantly different among treatments. The highest flavonoid content was observed upon treatment with 12.5 µg/mL IONPs, which generated an increase of 51.3% relative to the control. Similarly, fungicide treatment exceeded the control by 44.6%. NPs induce the generation and accumulation of anti-oxidants such as flavonoids, vitamin C, and carotenoids in plants, as a natural response against plant pathogens [102]. With respect to the vitamin C content, there was a 9.67–2.84% decrease when a lower-dose treatment (0.01 and 1.5 µg/mL) of IONPs was applied. The three highest doses of IONPs (10 and 15 µg/mL), including fungicide, significantly increased the vitamin C content in tomato fruit by 12.9, 14.5, 13.9, and 10.1%, respectively. Vitamin C is the most vital component of tomato fruit and plays a key role in preventing oxidative damage [96]. Quiterio-Gutiérrez et al*.* reported an increase in flavonoid and vitamin C content in tomato fruits upon application of selenium and copper NPs [102]. Proteins play a key role in fruit growth and quality, and tomato ripening is associated with the function of regulatory proteins involved in the initiation of ethylene biosynthesis [103, 104]. Total protein content significantly increased upon treatments at the highest concentrations of IONPs, reaching up to 28.1, 61.4, 66.2, 73.9, and 70.3% of the content upon control treatment, with 5–15 µg/mL of IONPs, respectively, while there was an increase of 69.6% upon fungicide treatment. However, the other treatments did not show a significant difference from the control. Zhao et al*.* observed increased protein content in cucumber fruits after the application of NPs [105].

## Conclusions

In the present study, we explored green-synthesized IONPs and demonstrated that these nanoscale materials have the potential to become a part of the disease management system. IONPs were synthesized by means of a green approach, while using spinach as the starting material and BC as a reducing/stabilizing agent. The microwave power was varied from 100 to 1000 W, to tune the properties of the resulting product. XRD results revealed the cubic to spherical magnetite (Fe_3_O_4_) phase of the NPs, with a super-paramagnetic nature at all of the microwave powers. XPS results also confirmed the binding energies of Fe 2p_3/2_ (710.9 eV) and Fe 2p_1/2_ (724.5 eV) of the Fe_3_O_4_ NPs synthesized using a microwave power of 1000 W. FTIR analysis confirmed the presence of a cubic magnetite (Fe_3_O_4_) phase at all of the microwave powers. IONPs showed strong anti-fungal activity against *F. oxysporum* at the highest concentrations. Exposure to IONPs not only inhibited fungal growth in *vitro*, but also managed Fusarium wilt in tomato in a pot bioassay. ROS generation, mycelium deformation, and DNA fragmentation were found due to the interaction between NPs and fungal cells and could be related to the intrinsic mechanism of the IONPs. The biosynthesized IONPs positively affected the plant growth parameters and fruit quality, by reducing the disease index. The current findings indicated that these nanomaterials have the potential to repress phyto-fungal pathogens, by improving plant resistance. Iron, which is a micronutrient, also enhanced tomato growth parameters by functioning as a nano-fertilizer. Thus, these outcomes paved the way for the employment of these small particles (NPs) in the agricultural industry, as an eco-friendly approach.

## Supplementary Information

**Additional file 1: Experiment S1.** Instrumentation for characterization of IONPs. **Experiment S2.** Suppressive activity of Iron-oxide nanoparticles on fungal spore germination. **Experiment S3.** Evaluating plant physiology and defensive enzymes. **Figure S1.** Crystallite size and dislocation density of IONPs. **Figure S2.** Comparison of dielectric constant **a** and tangent loss **b** by varying microwave powers at log f = 1.3 and log f = 5. **Figure S3.** Conductivity plot of **a** IONPs v/s frequency at various microwave powers and (b) IONPs v/s microwave powers at log f = 5 and log f = 7.3. **Figure S4.** Variation in saturation magnetization of green synthesized iron oxide nanoparticles. **Figure S5.** Surface morphology and size distribution analysis. **Figure S6.** Agarose gel electrophoretic analysis of *F. oxysporum* DNA treated with various concentrations of IONPs. **Figure S7.** Effect of various concentrations of IONPs on growth variables. **Figure S8.** Effect of various concentrations of IONPs on disease attributes. **Figure S9.** Influence of various concentrations of IONPs on photosynthetic pigments. **Figure S10.** Effect on vegetative growth (roots and shoots) of tomato-plant exposed to different concentrations of IONPs. **Table S1.** Comparing the antifungal effect of Iron-oxide nanoparticles (IONPs) synthesized at various microwave power (100–1000 W) on mycelial growth of *F. oxysporum* after seven days of incubation at 28 °C.

## Data Availability

All data generated or analyzed in this study are included in this published article and its Additional file 1.

## Abbreviations

IONPs: Iron oxide nanoparticles
ROS: Reactive oxygen species
XRD: X-Ray Diffractometer
FTIR: Fourier Transform Infra-red spectroscopy
VSM: Vibrating sample magnetometer
XPS: X-ray Photoelectron spectroscopy
SEM: Scanning electron microscope
TEM: Transmission electron microscope
DI: Deionized water (DI)
PDA: Potato dextrose agar
BC: Black coffee
H_2_DCFDA: Dichloro-dihydro-fluorescein-diacetate
PBS: Phosphate buffer saline
PI: Propidium iodide
DI: Disease incidence (DI)
CAT: Catalase
SOD: Superoxide-dismutase
POD: Peroxidase
APX: Ascorbate-peroxidase
GPX: Glutathione-peroxidase
