# Supplementary material for: Sustainable synthesis of microwave-assisted IONPs using Spinacia oleracea L. for control of fungal wilt by modulating the defense system in tomato plants
Source: J Nanobiotechnology. 2022 Jan 4;20:8. doi: 10.1186/s12951-021-01204-9 (PMC8725286; doi:10.1186/s12951-021-01204-9)
Supplement: Supplementary file 1 — Additional file 1: Experiment S1. Instrumentation for characterization of IONPs. Experiment S2. Suppressive activity of Iron-oxide nanoparticles on fungal spore germination. Experiment S3. Evaluating plant physiology and defensive enzymes. Figure S1. Crystallite size and dislocation density of IONPs. Figure S2. Comparison of dielectric constant a and tangent loss b by varying microwave powers at log f = 1.3 and log f = 5. Figure S3. Conductivity plot of a IONPs v/s frequency at various microwave powers and (b) IONPs v/s microwave powers at log f = 5 and log f = 7.3. Figure S4. Variation in saturation magnetization of green synthesized iron oxide nanoparticles. Figure S5. Surface morphology and size distribution analysis. Figure S6. Agarose gel electrophoretic analysis of F. oxysporum DNA treated with various concentrations of IONPs. Figure S7. Effect of various concentrations of IONPs on growth variables. Figure S8. Effect of various concentrations of IONPs on disease attributes. Figure S9. Influence of various concentrations of IONPs on photosynthetic pigments. Figure S10. Effect on vegetative growth (roots and shoots) of tomato-plant exposed to different concentrations of IONPs. Table S1. Comparing the antifungal effect of Iron-oxide nanoparticles (IONPs) synthesized at various microwave power (100–1000 W) on mycelial growth of F. oxysporum after seven days of incubation at 28 °C. [file 12951_2021_1204_MOESM1_ESM.docx]

**Additional file 1**

**Sustainable synthesis of microwave-assisted IONPs by using *Spinacia oleracea* L. for the control of fungal wilt by modulating defense system in tomato plants**

Hina Ashraf ^(1,2,3),^ Tehmina Anjum ^(2)^, Saira Riaz ^(3)^, Tanzeela Batool ^(3)^, Shahzad Naseem ^(3)^, Guihua Li ^(1) *^

*Corresponding Author: [*liguihua@gdaas.cn*](mailto:liguihua@gdaas.cn)

**Additional file 1 Experiments:**

**Experiment S1. Instrumentation for characterization of IONPs**

Green synthesized iron oxide nanoparticles (IONPs) were characterized using X-Ray Diffractometer (Bruker D8 Advance) with CuKα (1.5406 Å) radiations. FTIR analyses were performed by using Shimadzu IR Tracer -100 Fourier Transform Infra-red (FTIR) spectroscopy. The dielectric properties of the samples was determined by a 6500B (Wayne-Kerr) precision impedance analyzer whereas, the magnetic properties of nanoparticles were studied by using a vibrating sample magnetometer (VSM- Lake Shore’s 7407 ). X-ray Photoelectron spectroscopic (XPS) analyses were obtained by the ThermoFisher ESCALAB system. A scanning electron microscope (SEM-TESCAN-VegaLMU, 30.0KV) and Transmission electron microscope (TEM- JEOL 2010F) was used for investigating the morphology and size of nanoparticles.

**Experiment S2. Suppressive activity of Iron-oxide nanoparticles on fungal spore germination**

The concavity slide method was used to detect the suppressive activity of iron-oxide nanoparticles on spore germination of *F. oxysporum* [1]. Spores were obtained from the seven-day-old culture of *F. oxysporum* grown on potato dextrose broth, collected by centrifugation for 3 mins at 7000 rpm accompanied by twice washing with sterile distilled water. The concentration of spore-suspension was determined and calibrated to 1 x 10^7^ spore mL^-1^ by using a haemocytometer (Hausser Scientific, Horsham, PA, USA). Then, 100 µL aliquots of both spore suspension and IONPs was taken in Eppendorf to get series of aforesaid concentrations (0.01-15 µg/mL). Then approximately, 50 µL of blended spore suspension was dropped on a sterile glass slide and incubated at 28°C for six hours. Spore-suspension without incorporation of nanoparticles, served as the control, was placed on slides. The number of total spores and germinated-spores at four arbitrarily selected zones was observed in every slide under light-microscope whereas, images were taken by digital camera. Each treatment was replicated thrice including control. Lastly, the percentage of germinated spores were analyzed via, rate of an average number of germinated spores to a total number of spores.

**Experiment 3. Evaluating plant physiology and defensive enzymes**

Various physiological and biochemical variations in tomato plants were accessed on the 3^rd^ day after the second foliar-spray of various concentrations of nanoparticles. Total chlorophyll and carotenoid content in leaves (0.1 g) extract, prepared in 100% acetone was anticipated by ensuing the protocol by Pocock et al. [2]. The absorbance was taken for chlorophyll “a” and “b” at 664 nm and 647 nm and at 470 nm for carotenoid, analysed according to the formula derived by Wellburn et al. [3]. Total phenolic content, superoxide dismutase (SOD), peroxidases (POD), catalase (CAT), ascorbate-peroxidase (APX) and glutathione-peroxidase (GPX) activities in root and shoots of tomato plant were quantified according to the earlier described protocols [4,5]. Total protein content, vitamin C, lycopene and flavonoid content in tomato fruits were measured according to the protocols given by Lopez-Vargas et al. [6].

**Additional file 1 Figures:**





**Figure S1**. Crystallite size and dislocation density of IONPs synthesized using various microwave powers (100 W-1000W).







**Figure S2**. Comparison of dielectric constant (a) and tangent loss (b) by varying microwave powers at log *f* = 1.3 and log *f* = 5.







**Figure S3.** Conductivity plot of (a) IONPs v/s frequency at various microwave powers and (b) IONPs v/s microwave powers at log *f* =5 and log *f* = 7.3.

**

**

**Figure S4.** Variation in saturation magnetization of green synthesized iron oxide nanoparticles synthesized at various microwave powers (100 W- 1000 W).


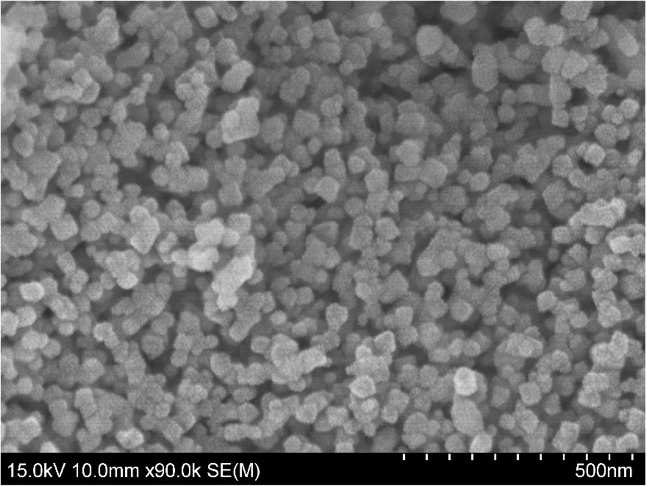

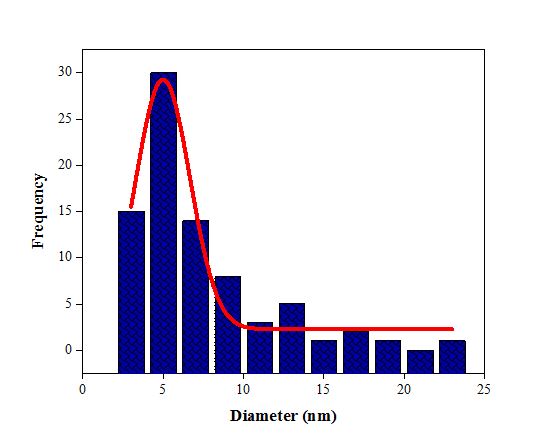

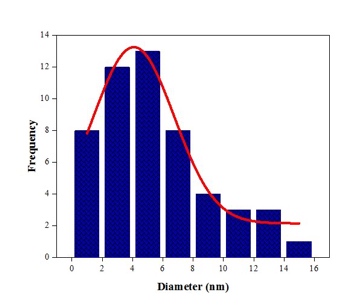

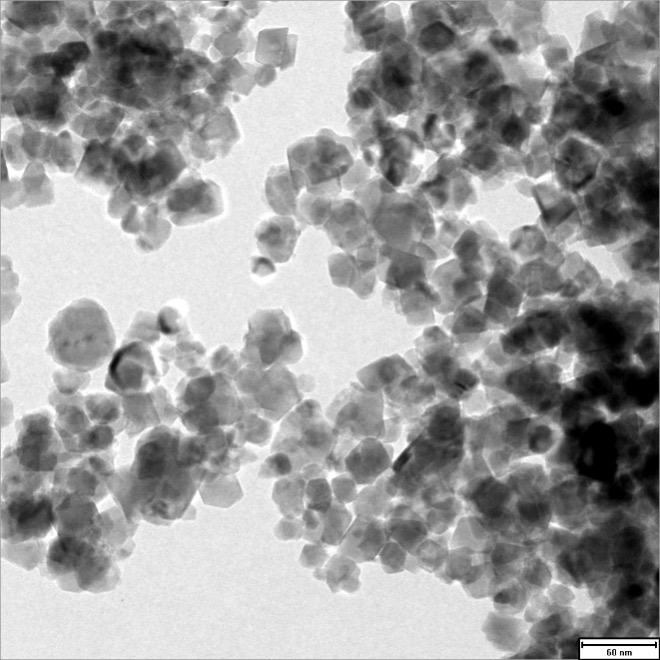


**Mean: 4.99 nm**

**Std. Dev: 0.17 nm**

**N: 80**

**Mean: 4.08 nm**

**Std. Dev: 0.19 nm**

**N: 52**

**(a)**

**(b)**

**(c)**

**(d)**

**Figure S5**. **Surface morphology and size distribution analysis:** SEM and TEM images (a & c) of IONPs synthesized at 1000 W microwave powers along with size distribution histograms (b& d).

#

#
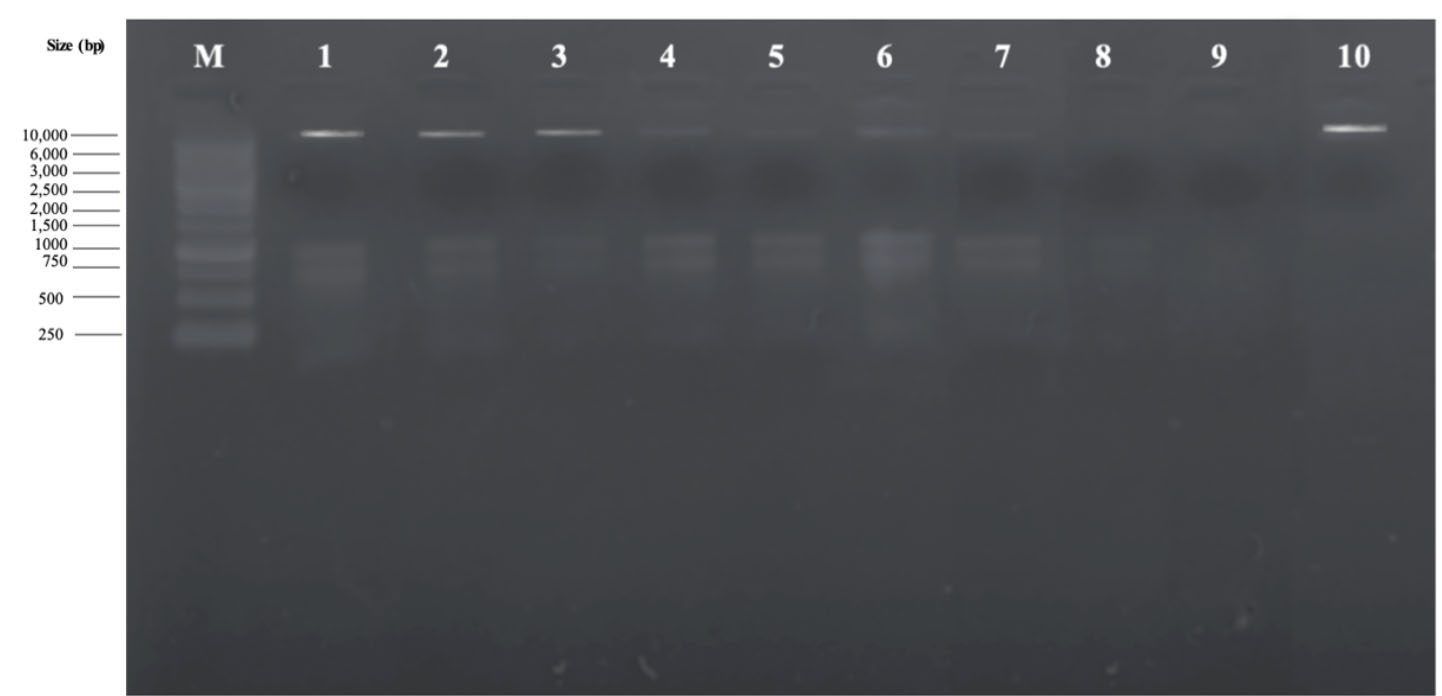


# Figure S6. Agarose gel electrophoretic analysis of *F. oxysporum* DNA treated with various concentrations of IONPs: Lane M: DNA Marker, Lane 1: 0.01 µg/mL, Lane 2: 0.5 µg/mL, Lane 3: 1.5 µg/mL, Lane 4: 2.5 µg/mL, Lane 5: 5 µg/mL, Lane 6: 7.5 µg/mL, Lane 7: 10 µg/mL, Lane 8: 12.5 µg/mL, Lane 9: 15 µg/mL, Lane 10: Control

**
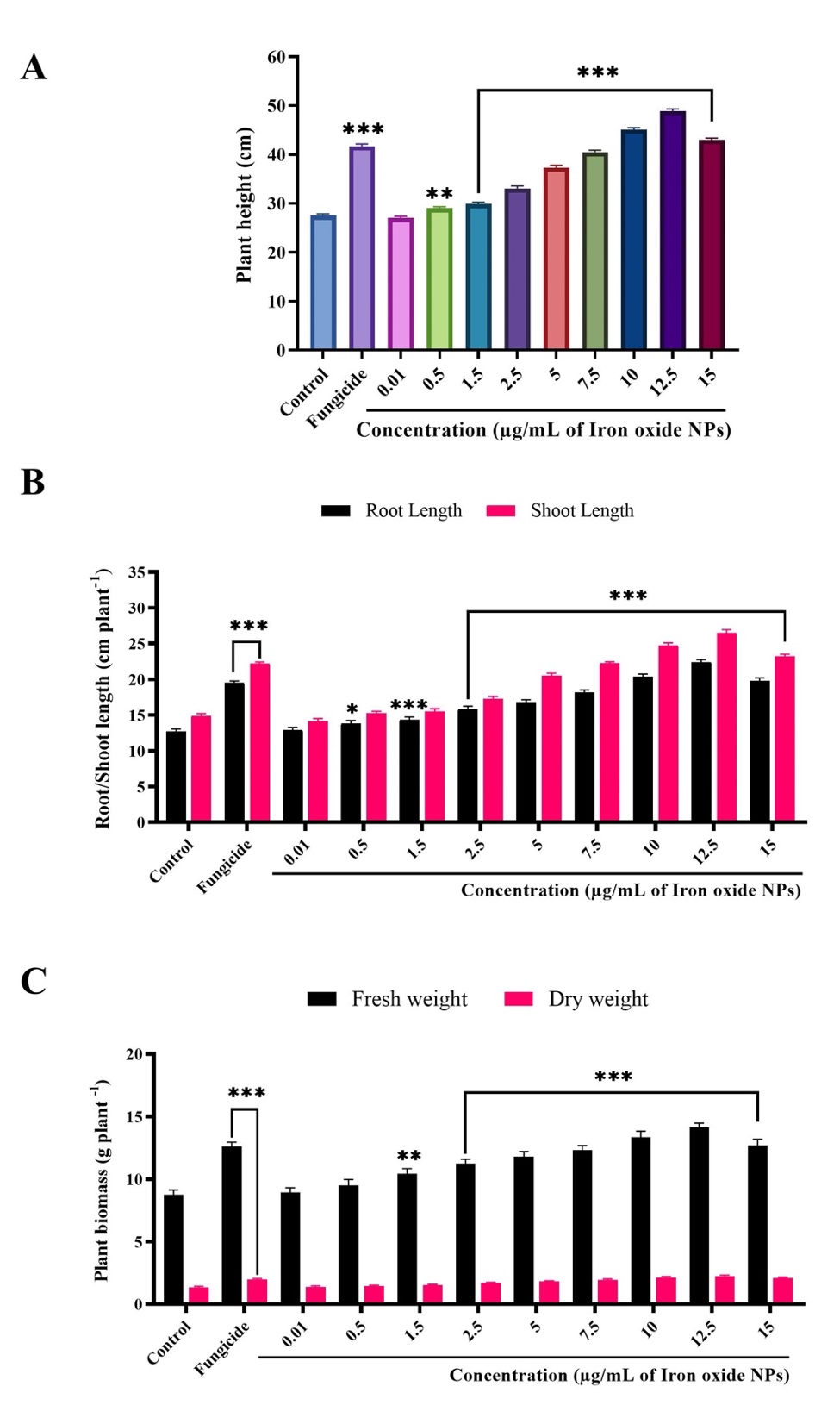
**

**Figure S7. Effect of various concentrations of IONPs on growth variables:** (A) plant height, (B) length (root & shoot) and (C) biomass (fresh & dry) of tomato plants infected with *F. oxysporum* under pot condition. Significant-difference (* P< 0.05; **P < 0.01; *** P < 0.001) among different concentrations of IONPs and control group performed by one-way-ANOVA at P < 0.05 and Tukey multiple comparisons analysis. Error bar represents a mean ± SD of five replicates.

**
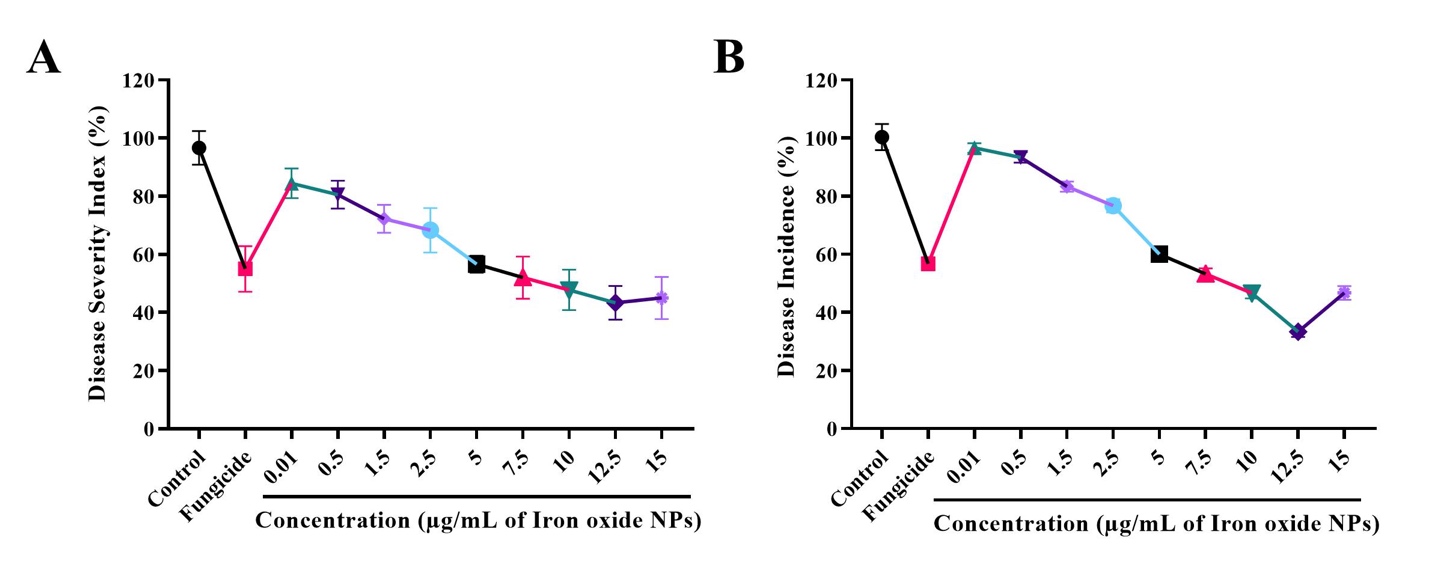
**

**Figure S8. Effect of various concentrations of IONPs on disease attributes** (A) disease incidence, (B) disease severity of tomato plants infected with *Fusarium oxysporum* under pot condition. Error bar represents a mean ± SD of five replicates.


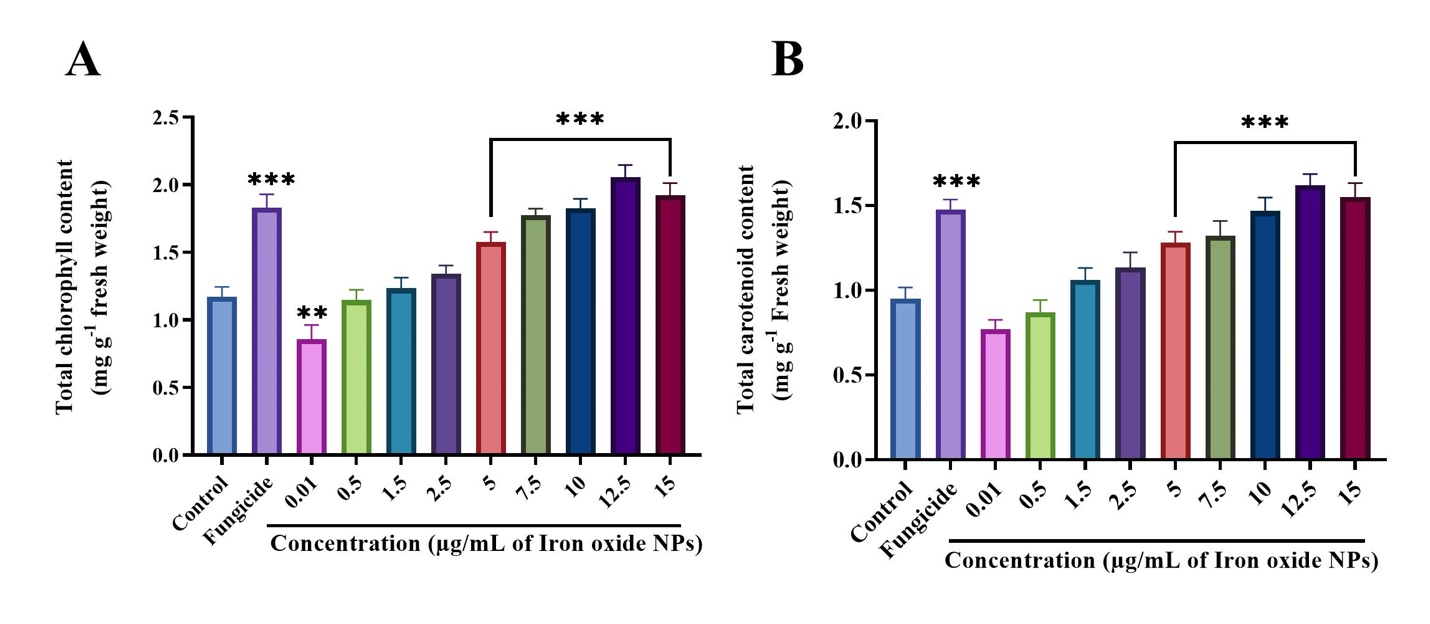

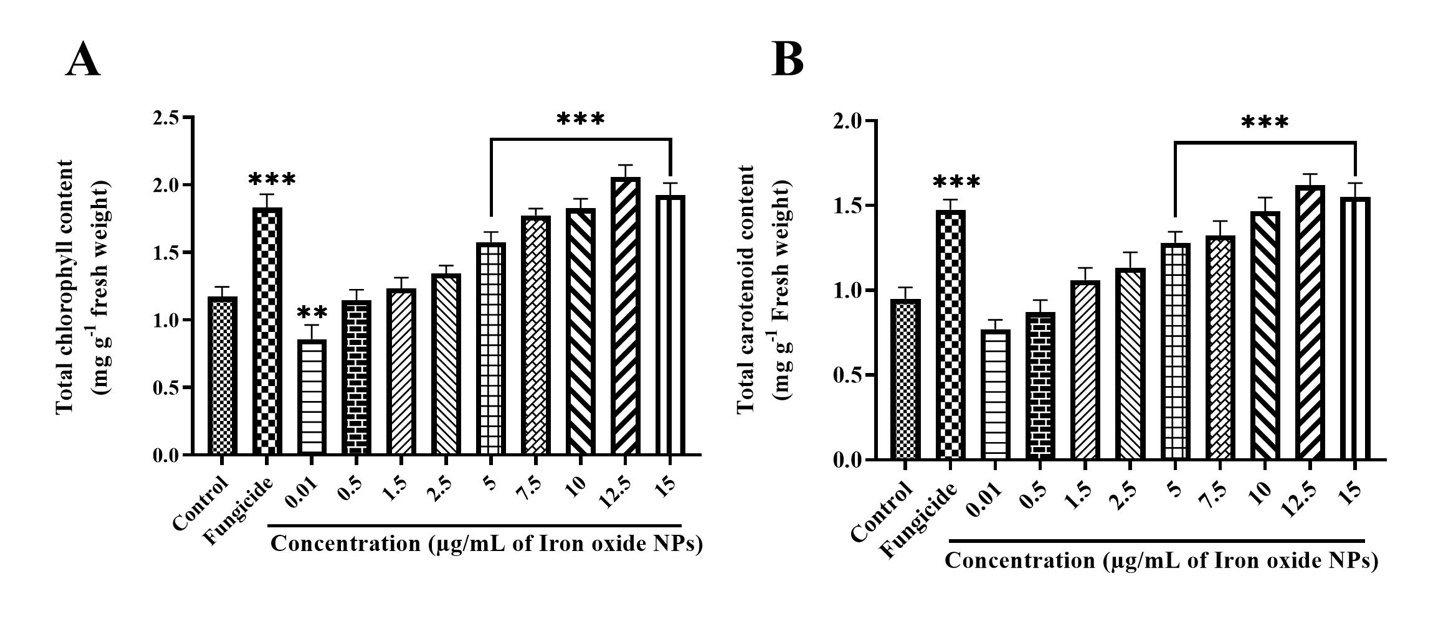


**Figure S9. Influence of various concentrations of IONPs on photosynthetic pigments:** (A) chlorophyll, (B) carotenoid content of tomato plants infected with *Fusarium oxysporum* under pot condition. Significant-difference (*P < 0.05; **P < 0.01; ***P < 0.001) among different concentrations of IONPs and control group performed by one-way-ANOVA at P < 0.05 and Tukey multiple comparisons analysis. Error bar represents a mean ± SD of five replicates.


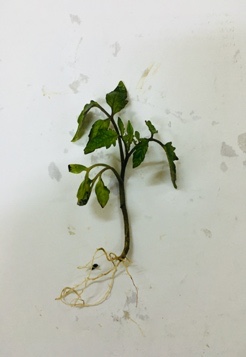

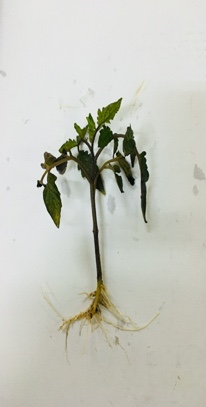

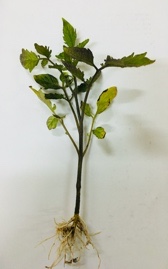

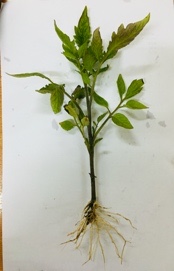

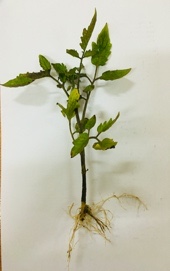

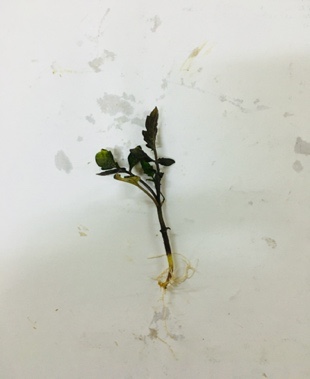

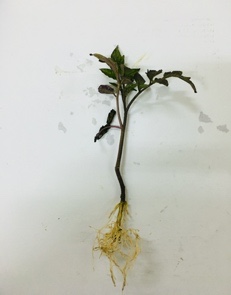

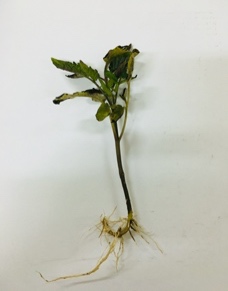

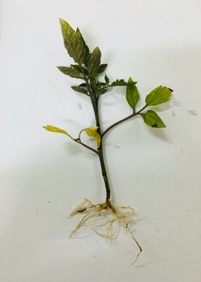

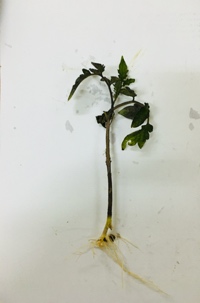

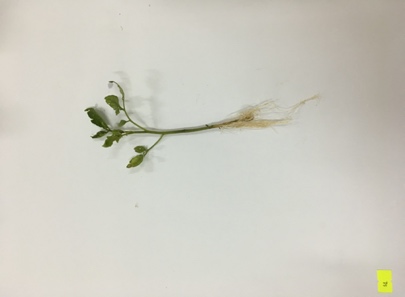


**A**

**C**

**B**

**D**

**E**

**F**

**G**

**H**

**J**

**I**

**K**

**Figure S10. Effect on vegetative growth (roots and shoots) of tomato-plant exposed to different concentrations of IONPs.** A: control (inoculated with *F. oxysporum* only), B-J various concentrations of IONPs (µg/mL), B: 0.01, C: 0.5, D: 1.5, E: 2.5, F: 5, G: 7.5, H: 10, I: 12.5, J: 15 and K: fungicide treatment.

**Additional file 1 Tables:**

**Table S1:** Comparing the antifungal effect of Iron-oxide nanoparticles (IONPs) synthesized at various microwave power (100 W- 1000 W) on mycelial growth of *F. oxysporum* after seven days of incubation at 28ºC.

| Sr no. | Microwave powers (W) | * Percentage inhibition zone (%) |
| --- | --- | --- |
| 1. | 100 | 85.6 |
| 2. | 200 | 87.9 |
| 3. | 300 | 88.9 |
| 4. | 400 | 90.5 |
| 5. | 500 | 92.1 |
| 6. | 600 | 92.5 |
| 7. | 700 | 93.3 |
| 8. | 800 | 93.8 |
| 9. | 900 | 94.9 |
| 10. | 1000 | 95.5 |

*A concentration of 15 µg/ mL from each solution of IONPs was used to estimate the inhibition zone in parallel to the control treatment.

**Additional file 1 References:**

1. Chen JN, Peng H, Wang XP, Shao F, Yuan ZD, Han HY. Graphene Oxide Exhibits Broad-Spectrum Antimicrobial Activity Against Bacterial Phytopathogens and Fungal Conidia by Intertwining and Membrane Perturbation. Nanoscale. 2014: 6: 1879–1889.
2. Pocock T, Król M, Huner NP. The Determination and Quantification of Photosynthetic Pigments by Reverse Phase High-Performance Liquid Chromatography, Thin-Layer Chromatography, and Spectrophotometry. Methods Mol Biol. 2004;274:137–148.
3. Wellburn AR. The Spectral Determination of Chlorophylls a and b, as well as Total Carotenoids, using Various Solvents with Spectrophotometers of Different Resolution. J Plant Physiol. 1994; 144:307–313.
4. Moradbeygi H, Jamei R, Heidari R, Darvishzadeh R. Investigating the Enzymatic and Non-Enzymatic Antioxidant Defense by Applying Iron Oxide Nanoparticles in *Dracocephalum moldavica* L. Plant under Salinity Stress. Sci Hortic. 2020;272: 109537.
5. Chahardoli A, Karimi N, Ma X, Qalekhani F*.* Effects of Engineered Aluminum and Nickel Oxide Nanoparticles on the Growth and Antioxidant Defense Systems of *Nigella arvensis* L. Sci Rep. 2020; 10: 3847.
6. López-Vargas E, Ortega-Ortíz H, Cadenas-Pliego G, de Alba Romenus K, Cabrera de la Fuente M, Benavides-Mendoza A, Juárez-Maldonado A. Foliar Application of Copper Nanoparticles Increases the Fruit Quality and the Content of Bioactive Compounds in Tomatoes. Appl Sci. 2018; 8:1020.
